# QiDiTangShen Granules Activate Renal Nutrient-Sensing Associated Autophagy in db/db Mice

**DOI:** 10.3389/fphys.2019.01224

**Published:** 2019-10-01

**Authors:** Xiangming Wang, Li Zhao, Amrendra K. Ajay, Baihai Jiao, Xianhui Zhang, Chunguo Wang, Xue Gao, Zhongyu Yuan, Hongfang Liu, Wei Jing Liu

**Affiliations:** ^1^Department of Endocrinology and Nephrology, Renal Research Institute of Beijing University of Chinese Medicine, Dongzhimen Hospital Affiliated to Beijing University of Chinese Medicine, Beijing, China; ^2^Key Laboratory of Chinese Internal Medicine of Ministry of Education and Beijing, Dongzhimen Hospital Affiliated to Beijing University of Chinese Medicine, Beijing, China; ^3^Department of Medicine, Brigham and Women’s Hospital, Harvard Medical School, Boston, MA, United States; ^4^Department of Medicine, University of Connecticut Health Center, Farmington, CT, United States; ^5^Health Management Center, Dongzhimen Hospital Affiliated to Beijing University of Chinese Medicine, Beijing, China; ^6^Beijing Research Institute of Chinese Medicine, Beijing University of Chinese Medicine, Beijing, China

**Keywords:** diabetic nephropathy, proteinuria, renal injury, Chinese medicine, nutrient-sensing signal, autophagy

## Abstract

QiDiTangShen granules (QDTS) have been proven to reduce the proteinuria in patients with diabetic nephropathy (DN) effectively. The present study was aimed to investigate the mechanism underlying QDTS’s renoprotection. The main components of QDTS were identified by ultra-high liquid chromatography-tandem mass spectrometry and pharmacological databases, among which active components were screened by oral bioavailability and drug-likeness. Their regulation on autophagy-related nutrient-sensing signal molecules (AMPK, SIRT1, and mTOR) was retrieved and analyzed through the Pubmed database. Then, db/db mice were randomly divided into three groups (model control, valsartan and QDTS), and given intragastric administration for 12 weeks, separately. Fasting and random blood glucose, body weight, urinary albumin excretion (UAE) and injury markers of liver and kidney were investigated to evaluate the effects and safety. Renal histological lesions were assessed, and the expressions of proteins related to nutrient-sensing signals and autophagy were investigated. Thirteen active components were screened from 78 components identified. Over half the components had already been reported to improve nutrient-sensing signals. QDTS significantly reduced UAE, ameliorated mesangial matrix deposition, alleviate the expression of protein and mRNA of TGF-β, α-SMA, and Col I, as well as improved the quality of mitochondria and the number of autophagic vesicles of renal tubular cells although the blood glucose was not decreased in db/db mice. Compared to the db/db group, the expression of the autophagy-inducible protein (Atg14 and Beclin1) and microtubule-associated protein 1 light chain 3-II (LC3-II) were up-regulated, autophagic substrate transporter p62 was down-regulated in QDTS group. It was also found that the expression of SIRT1 and the proportion of p-AMPK (thr172)/AMPK were increased, while the p-mTOR (ser2448)/mTOR ratio was decreased after QDTS treatment in db/db mice, which was consistent with the effect of its active ingredients on the nutrient-sensing signal pathway as reported previously. Therefore, QDTS may prevent the progression of DN by offering the anti-fibrotic effect. The renoprotection is probably attributable to the regulation of nutrient-sensing signal pathways, which activates autophagy.

## Introduction

Diabetic nephropathy (DN) has been the leading cause of patients with end-stage renal disease requiring dialysis or renal replacement therapy (Gross et al., 2005). The International Diabetes Federation survey (2017) showed that people diagnosed with diabetes and clinical kidney disease have 50% more medical expenses ($6,826/year) than those diagnosed with diabetes without clinical kidney disease (Federation, 2017). Currently, the treatment of DN mainly focuses on controlling blood glucose, blood lipids and blood pressure, and the application of vasoactive drugs (angiotensin-converting enzyme inhibitors and angiotensin receptor blockers, ACEI and ARB), *etc*. To a certain extent, these measures can delay the progression of diabetic kidney disease, but they cannot completely block the process forwards renal failure (Sulaiman, 2019). Therefore, complementary and alternative medicine, especially traditional Chinese medicine, is striving to find more promising therapeutic strategies.

Proteinuria is an independent risk factor and an important diagnostic and post-treatment evaluation marker for DN (Kishore et al., 2017). QiDiTangShen granules (QDTS), derived from the theory ‘filling the renal essence to treat kidney diseases’ proposed by Jingyue Zhang, a famous doctor of Ming Dynasty in China (Liu and Zhang, 2016), were formulated on the basis of more than 10 years’ clinical practice. It has been proven to reduce proteinuria of DN effectively independent of blood glucose control (Han et al., 2014; Gao et al., 2018). The mechanism of the renoprotective effect of QDTS remains to be clarified further.

Autophagy, an ancient regulation process that cells recognize and degrade damaged proteins, aging organelles and invading pathogens under dystrophic and various stress stimuli, then release products degraded for reuse to maintain cell homeostasis, is reserved during biological evolution (Mizushima and Komatsu, 2011). Emerging evidence suggested that renal autophagic suppression under the excessive-nutrients state of diabetes results in the lesions of renal histopathology in DN, such as not only accumulation of intracellular aging or damaged proteins and organelles both aroused by advanced glycation end products (AGEs) (Takahashi et al., 2017), but also accumulation of extracellular matrix (ECM) components and tubulointerstitial fibrosis, *etc.* (Song et al., 2017; Yang et al., 2018). Therefore, autophagy is considered a new promising therapeutic target for the treatment of DN (Kitada et al., 2017; Sakai et al., 2019).

The autophagic activity of cells is regulated by autophagy-related nutrient-sensing signaling pathways, such as AMP-activated protein kinase (AMPK), the mammalian target of rapamycin (mTOR) and sirtuins 1 (SIRT1), *etc*. (Kume et al., 2012). *Astragalus membranaceus*, as the core component of QDTS, up-regulated the AMPK signaling pathway, which was altered under the excessive supply of nutrients (Zhang et al., 2018). Therefore, we estimated that QDTS might ameliorate renal lesions by alleviating renal autophagic suppression through the regulation of nutrient-sensing signals such as AMPK, mTOR, and SIRT1. Firstly, active ingredients within QDTS were identified and screened out by ultra-high performance liquid chromatography-tandem mass spectrometry (UHPLC-MS) and pharmacological databases. The effects of these ingredients on the above-mentioned nutrient-sensing molecules were analyzed in succession. Secondly, we tried to confirm whether the altered nutrient-sensing signaling pathways, suppressed autophagic activity, and renal injury under the state of DN were ameliorated by QDTS.

## Materials and Methods

### Medicines and Reagents

QiDiTangShen granules, whose patent is currently under review (Patent Accepting No. 201410436459.6) from the State Food and Drug Administration of China, contains seven Chinese herbs, including Astragali Radix (*Astragalus membranaceus* Fisch., Inner Mongolia Autonomous Region), Rehmanniae radix praeparata (*Rehmannia glutinosa* Libosch., Henan Province), Euryale Semen (*Euryale ferox* Salisb., Hunan Province), Sophorae Tonkinensis Radix et Rhizoma(*Sophora tonkinensis* Gagnep., Zhejiang Province), Hirudo (*Whitemania pigra* Whitman, Guangdong Province), Rhei Radix et Rhizoma (*Rheum palmatum* L. Qinghai Province) and Baihuasheshecao (*Hedyotis diffusa* Willd., Fujian Province). The extract of QDTS was provided by Zhuozhoudongle Pharmaceutical Co., Ltd. (Hebei, China). The doses of QDTS and valsartan (Beijing Novartis Pharmaceutical Co., Ltd., Beijing, China) for mice were 3.37 g/kg/day and 10.29 mg/kg/day, respectively, which were acquired at a ratio with the regular human adult dosage (Gao et al., 2018).

Acetonitrile, analytical grade methanol, and formic acid (HPLC grade) were purchased from Thermo Scientific (Waltham, MA, United States). Ultra-pure water (18.2 MΩ⋅cm) was from a Milli-Q system (Millipore, Bedford, MA, United States). Polytetrafluoroethylene (PTFE) membranes of 0.45 micron used for processing samples were from ANPEL (Shanghai, China).

### UHPLC-MS Analysis

The extract of QDTS was dissolved within the solution of methanol and water (6:4, v/v) at 1:20, sonicated for 40 min and centrifuged at 3000 rpm for 5 min. The supernatant was transferred and filtered through a 0.45 microns PTFE membrane, and then stored at 4°C for analysis.

Chromatographic analysis was performed with a UHPLC System (Thermo Scientific Dionex Utimate 3000, Thermo Corporation, United States). The conditions were as follows, column: AQUITY UPLC C18 column (2.1 mm × 100 mm, 1.7 μm); mobile phase: 0.1% aqueous formic acid (A), acetonitrile solution (B); gradient elution conditions: 0 ∼ 3 min (5% ∼ 5% B), 3 ∼ 45 min (5% ∼ 75% B), 45 ∼ 45.1 min (75 ∼ 5% B), 45.1 ∼ 50 min (5% ∼ 5% B); flow speed: 0.3 ml/min; injection volume: 2 μl; column temperature: 35°C.

Mass spectrometry was performed with a hybrid linear ion trap–FTICR (7-Tesla) mass spectrometer (LTQ-Oribitrap XL, Thermo Scientific, United States) equipped with an electrospray ionization source and operated with the Xcalibur (version 2.1) data acquisition software. The experimental conditions were as follows, positive ion detection mode: HESI ion source, ion source temperature: 350°C, ionization source voltage: 4 KV, capillary voltage: 35 V, tube lens voltage: 110 V, sheath gas and auxiliary gas: high purity nitrogen (purity > 99.99%), sheath gas flow rate: 40 arb, auxiliary gas flow rate: 20 arb; negative ion detection mode: HESI ion source, ion source temperature: 350°C, ionization source voltage: 3 KV, capillary voltage: 35 V, tube lens voltage: 110 V, sheath gas and the auxiliary gas: high purity nitrogen (purity > 99.99%), sheath gas flow rate: 30 arb, and auxiliary gas flow rate: 10 arb. Data of the positive and negative ion were collected by the method of Fourier transform high-resolution full-scan (TF, Full scan, Resolution 30000). Data-dependent acquisition (MS3) was performed by the method of collision-induced dissociation.

### Identification and Screening of Pharmaceutical Components

The total ions chromatography was obtained in the positive and negative ion modes. The components were presumed according to the retention time of chemical components detected in UHPLC-MS, high resolution accurate molecular weight, MS multi-level fragment information, combined with extracted ion flow diagram and standard information of Scifinder database, etc., which was used in our previous research (Wang et al., 2018). Each component identified was checked and revalidated in the PubChem database and Chemical Abstracts Service (CAS) for the number of PubChem CID or CAS. According to the oral bioavailability (OB ≥ 30%) and drug-likeness (DL ≥ 0.18), the active components were screened out from the Traditional Chinese Medicine Systems Pharmacology Database and Analysis Platform (TCMSP) (Li et al., 2015). The articles referred to the correlation between each effective ingredient and the nutrient-sensing signal molecules (AMPK, mTOR, and SIRT1) were retrieved and analyzed on the Pubmed database.

### Animals

The protocol of this research was approved by the Animal Welfare and Ethics Review Committee of Dongzhimen Hospital affiliated to Beijing University of Chinese Medicine (certificate number: 17-06) and was conducted in accordance with Management Regulations of Beijing Laboratory Animal (2004 Revision).

The db/db mice used in this experiment, 8 weeks old, 20 ± 3 g, were purchased from Beijing Vital River Laboratory Animal Technology Co., Ltd. (certificate number: SCXK [Beijing]2012-0001). Wild type C57BL/6J mice were used as normal control. All the mice were kept in the barrier-level animal room of Dongzhimen Hospital affiliated to Beijing University of Chinese Medicine (certificate number: SYXK[Beijing] 2015-0001). The conditions were as follows, switch light cycle 12/12 h, temperature 22–24°C, humidity 45–50%, and the litter replaced once a day.

At 12 weeks old, consecutive twice random blood glucose levels higher than 13.5 mmol/L were model criteria for db/db mice for further research. They were randomly divided into three groups (*n* = 12) by random numbers, named as db/db group (model control), db/db + valsartan (db/db + V) and db/db + QDTS (db/db + Q), separately. Intragastric administration with 0.5 ml solutions (0.85% saline for normal group and model group, valsartan for db/db + V and QDTS for db/db + Q, respectively) was started at the 13th week, once a day. All mice were weighed once a week. Random blood glucose and fasting blood glucose of tail blood were measured every 2 weeks. In the case of water supplied only, the volume of 12 h urine was collected to evaluate the level of Urinary albumin excretion (UAE) every 2 weeks. In the 25th week, blood from the left eye socket was collected for the detection of biochemical indicators such as serum creatinine, urea nitrogen, aspartate aminotransferase (AST) and alanine aminotransferase (ALT). The mice were then sacrificed by cervical dislocation. The right kidney was weighed to calculate the ratio of kidney weight to body weight. Blocks of kidney for pathology, transmission electron microscopy, and molecular biology testing were stored in 4% paraformaldehyde, 2.5% glutaraldehyde solution and refrigerators (−80°C), respectively.

### Detection of Pharmacodynamic Items

Random and fasting blood glucose was measured by OneTouch Ultra Glucometer (Johnson & Johnson, United States). Urinary albumin was detected by mice albumin ELISA Kit (R&D Systems, Minneapolis, MN, United States). Serum creatinine, blood urea nitrogen, serum AST, and ALT were analyzed by the automatic biochemical analyzer (Olympus AU480, Japan). The methods above were described in detail in our previous article (Gao et al., 2018).

### Renal Histological Examination

The tissue was fixed with 4% paraformaldehyde for 72 h and then rinsed with running water for 12 h. The process from dehydration to penetration with paraffin was according to standard procedures by a fully automatic closed tissue dewatering machine. Then, samples were processed as follows, embedded within paraffin and stored in a refrigerator at 4°C, cut into paraffin sections with 3 micron thicknesses, baked at 62°C for 90 min, deparaffined three times within xylene, each time for 12 min, respectively, dexylened by downgrading gradient ethanol solutions, each 3 min, and finally immersed into deionized water for subsequent staining.

### Hematoxylin and Eosin (HE) Staining

The sections above were dip-stained with hematoxylin dye solution for 5 min, rinsed with distilled water; differentiated with 1% hydrochloric acid alcohol solution for 3 s, rinsed with distilled water for 10–20 s, diluted with ammonia (PH = 8.0), rinsed with distilled water, then stained within 0.5% eosin solution for 3 min and dehydrated by the ascending gradient concentration of alcohol solutions for 3 min each time. They were made transparent with xylene three times for 10 min each time and covered with coverslips and natural resin.

### Masson Staining

The sections prepared were stained with Masson’s trichrome staining kit (Nanjing Jiancheng Biological Reagent Co., Ltd., Nanjing, China). They were firstly stained with nuclear dyeing reagent I for 6 min at room temperature, rinsed with distilled water; then treated with cytoplasm dyeing solution II for 30 s, rinsed with distilled water, then soaked with the color separation solution III for 8 min, then discard solution III and soaked with the reagent IV for 5 min and 30 s directly. Finally, they were dehydrated, made transparent with xylene, and sealed with natural resin.

### Periodic Schiff-Methenamine Silver (PASM) Staining

The sections were stained with the PASM kit (Zhuhai Baso Biotechnology Co., Ltd., Zhuhai, China). The powder of hexamethylenetetramine silver was fully dissolved by the borax solution in a clean dyeing tank and was preheated to 62°C by water bath firstly. The sections above were soaked in a periodic acid solution for 15 min, rinsed with running water for 6 min, then immersed in distilled water for 3 min and immersed in a dyeing tank at 62°C for 15 min. The reaction was terminated with distilled water, washed with running water for 3 min and then rewashed with double-distilled water for 5 min, reacted with sodium thiosulfate solution for 3 min, rinsed with running water for 2 min and then immersed in double-distilled water, dehydrated, made transparent with xylene and sealed with natural resin at last.

### Immunohistochemical Staining (IHC)

The kidney sections treated above were incubated with citrate antigen retrieval solution (pH = 6.0) at 95°C for 20 min and returned to room temperature naturally. The immunohistochemistry kit (SAP-9100, HistostainTM - SAP Kit, ZSBIO company, Beijing, China) was used according to standard procedures. Sections were incubated with anti-TGF-β1 (1: 100, ab92486, Abcam), anti-Col I (1:100, ab34710, Abcam) and anti-α-SMA antibody (1:100, ab32575, Abcam) overnight at 4°C, separately, and then incubated with horseradish peroxidase-conjugated anti-rabbit secondary antibody. After development with diaminobenzidine, the nuclear were stained with hematoxylin. Ten fields per sample were taken randomly with an optical microscope (400x). The cumulative optical density and area of the area of interesting were collected and calculated using Imagine pro plus, and the values of average optical density were calculated by Imagine Pro Plus software for statistical analysis.

### Quantitative Reverse Transcriptase Polymerase Chain Reaction (qRT-PCR)

Total RNA of renal tissues was extracted with TRIzol reagent (DP405-02, Tiangen Biotech Co., Ltd., Beijing, China) according to the manufacturer’s standard protocols. cDNA was synthesized using the First Strand cDNA Synthesis Kit (KR118, Tiangen Biotech Co., Ltd., Beijing, China). Amplification reactions were administrated using Talent qPCR kit (FP209, Tiangen Biotech Co., Ltd., Beijing, China). qPCRs were run in a 3,000 Fast Real-Time PCR System (Stratagene Mx3000P, Agilent Technologies Inc., Santa Clara, CA, United States). The 2^–ΔΔCt^ methods were used for relative mRNA quantification with β-actin as an internal control. Three replicates for each set of samples. Primer sequences are listed in Table 1.

**TABLE 1:** Primer information.

**Gene symbol**	**Primers**	**Sequence (5′→3′)**	**Gene ID**	**Product Length (bps)**
TGF-β	Forward	CGCAACAACGCCATCTATGA	21803	204
	Reverse	ACCAAGGTAACGCCAGGAAT		
Col I	Forward	ACAGGCGAACAAGGTGACA	192261	82
	Reverse	GAGAACCAGGAGAACCAGGAG		
α-SMA	Forward	GAACACGGCATCATCACCAA	11475	410
	Reverse	ATCTCACGCTCGGCAGTAG		
β-actin	Forward	AGAGGAGGAGGAGGAGAAGG	11461	280
	Reverse	CAGCCGAAGGACGAGGTAA		

### Western Blot

Five different samples were randomly selected from each group, and all results were repeated three times. The whole process of protein extraction was carried out on the ice. Samples were weighed and soaked into the RIPA Lysis Buffer (at 1:10) containing protease inhibitors (1:50, P1265, Applygen, Beijing, China) and protein phosphorylase inhibitors (1:100, P1260, Applygen, Beijing, China). The block of kidney tissue was cut into pieces by the sterile scissors and then were shattered intermittently by ultrasound for 2 min. After centrifugation at 15000 rpm at 4°C for 20 min, the supernatant was taken for measurement at 562 nm by the BCA method, and the protein concentration was quantified. After being diluted with loading buffer (5x), the protein extraction solution was being cooked at 95°C for 15 min, then divided and stored in a refrigerator (−80°C). Proteins with general molecular weight were electrophoresed in a 10% polyacrylamide gel, but for mTOR with molecular weight 289 KD, 8% Polyacrylamide gel and specific electroformed solution with a formaldehyde concentration of 10% were used. Proteins with molecular weight no more than 30 KD were treated with a 12% polyacrylamide gel. The voltage of the electrophoresis device and the current of the electrophoresis system are adjusted according to the molecular weight appropriately. The protein was transferred onto a cellulose acetate membrane with a diameter of 0.2 μm and then was blocked with 5% bovine serum albumin (phosphorylated proteins) or skimmed milk (Non-phosphorylated proteins). The first antibody was incubated on Shaker for overnight at 4°C. The membrane was washed with TBST (Tris-HCl, NaCl, and Tween20) for 10 min 3 times. The secondary antibody (diluted 1:5000 with 5% BSA or skimmed milk, SA00001-2, Proteintech, Wuhan, China) was incubated on a shaker at room temperature for an hour. Exposure of film was carried out with a luminescent liquid (SD239828, Thermo Fisher Scientific Co., Lot, Rockford, IL, United States) in a dark room. The value of photo densitometry of bands was measured with Imagine J. The antibodies used were as follows: SIRT1 (2977886, Millipore, Germany). mTOR (2972), p-mTOR Ser2448 (2971), phosphor-AMPKα (Thr172) (2535) and microtubule-associated protein 1 light chain 3-II (LC3-II) (4108) antibody (Cell Signal Technology, Inc., United States); autophagy-related protein 14 (Atg14) (ab139727), Beclin1 (ab207612) and SQSTM/p62 (ab109012) antibody (Abcam, Cambridge, United Kingdom); AMPK (66536) and secondary antibody (SA00001-2, 1:5000, Proteintech, Rosemont, IL, United States), β-actin (1:4000, C1828, Applygen, Beijing, China), all antibody were diluted by 1:1000 unless otherwise stated.

### Immunofluorescence

Five samples of each group were deparaffined to water routinely, and then incubated with citrate antigen retrieval solution (pH = 6.0) at 95°C for 20 min and returned to room temperature naturally. Slips were washed three times by phosphate-buffered saline (PBS, pH = 7.2–7.4), and were dealt with 0.2% PBST (Triton X-100 diluted with PBS at 1:500) to improve cell membrane permeability for 10 min, and blocked with 2% fetal bovine serum at 37°C for an hour, and then incubated with the primary antibody (ab51520, Abcam, Cambridge, United Kingdom, diluted at 1:500 with 0.2% PBST) at 4°C overnight. Slips were equilibrated for an hour at room temperature the next morning, and washed five times with PBS, 3 min each time, and then incubated with the secondary antibody (A-212206, Thermo Fisher, Waltham, MA, United States, 1:250 diluted with 0.2% PBST) at room temperature for 2 h in a dark humidified chamber, and then washed five times with PBS. At last, the nuclei were stained by DAPI with an anti-quenching agent. Samples were covered with coverslips. Ten fields of each sample were grasped randomly under a fluorescence microscope at 488 and 570 nm, respectively. Images were dealt with at the unified parameters and encoded with a new number, and then evaluated by three professionals for blind evaluation as described previously (Liu et al., 2012). The averages of scores were acquired for statistical analysis.

### Transmission Electron Microscopy (TEM)

Kidneys (three different samples of each group) were cut through the renal hilum longitudinally, and then cut into rectangular strips with a thickness of about 1 mm, fixed in 2.5% glutaraldehyde solution for 4 h (4°C), and rinsed four times with a 0.1 mol/L phosphate buffer, 15 min each time. Fixed with 1% citrate solution for 2 h and rinsed twice with 0.1 mol/L phosphate buffer for five min each time. 50, 70, 90, and 100% gradient acetone were needed to dehydrate samples for 15 min each. The samples were separately infiltrated with different ratios of 100% acetone and fatty trees (in a ratio of 1:1 and 2:1) for 2 h, respectively, and then infiltrated with pure resin overnight. They were embedded with Epon 812 resin, oven polymerization (37°C for 12 h, 45°C for 12 h, and 60°C for 48 h), made Ultra-thin sections with a thickness of 60 ∼ 70 nm (microtome model: Ultracut R, Leica) and stained with uranium acetate and lead nitrate. The fields of view were shot at 40000 and 70000 times (TEM model: Tecnai G2 Spirit Bio TWIN, FEI).

### Statistical Analysis

The data were expressed by means ± standard deviation. The normal distribution of the data was evaluated using the one-sample Kolmogorov–Smirnov test. Multiple comparisons were performed using the one-way ANOVA test. Data with equal variances were analyzed by the method of least significant difference. Data having unequal variances were analyzed by Dunnett’s T3. Statistical analyze was performed with SPSS 17.0 software, and *p*-value less than 0.05 was considered to have a statistical significance of groups.

## Results

### The Components Within QDTS Granules Might Ameliorate Nutrient-Sensing Signal Molecules

The mass spectrometry of 78 components with higher relative abundance was shown in the Supplementary Figure S1. The detailed information, including the name, molecular formula, molecular mass, characteristics of MS, source, OB, DL and the number of PubChem CID/CAS, were described in the Supplementary Table S1. The mass spectrograms, fragmentation pathway and chemical structural formula of Kaempferol, Bioquercetin and Astragaloside IV, as examples of the deriving process of all components, were shown in the Supplementary Figure S2.

According to pharmacokinetic characteristics (OB ≥ 30% and DL ≥ 0.18), 13 active components were screened out from the TCMSP database (Table 2). All of them had been included in the PubChem database and Chemical Abstracts Service (CAS). Beyond half components had been reported to ameliorate the three autophagy-related nutrient-sensing signal molecules AMPK (8/13), mTOR(7/13) and SIRT1(5/13) (Table 2).

**TABLE 2:** Active components of QDTS granules and their regulation on nutrient-sensing molecules.

**Number**	**Active components**	**Source**	**OB (%) and DL**	**Number of PubChem CID/CAS**	**Articles related to the activation of AMPK**	**Articles related to the regulation of mTOR**	**Articles related to the activation of SIRT1**
1	Formononetin	*Astragalus membranaceus*	69.67/0.21	5280378	Gautam et al., 2017	Yao et al., 2019	Hwang et al., 2018
2	Kaempferol	*Astragalus membranaceus*	41.88/0.24	5280863	Varshney et al., 2018	Che et al., 2017	Guo et al., 2015
3	Luteolin	*Astragalus membranaceus*	36.16/0.39	5280445	Wang Q. et al., 2017	Wang Q. et al., 2017	Rafacho et al., 2015
4	Isovitexin	*Herba hedyotis*	31.29/0.72	162350	–	–	–
5	Kumatakenin	*Astragalus membranaceus*	50.83/0.29	5318869	–	–	–
6	Calycosin	*Rheum officinale*	47.75/0.24	5280448	Han et al., 2018	Sun et al., 2018	–
7	Kaempferide	*Astragalus membranaceus*	73.41/0.28	5281666	–	–	–
8	Rhein	*Rheum officinale*	47.07/0.28	10168	Tu et al., 2017	Wu et al., 2017	Chen et al., 2015
9	Alizarin	*Rheum officinale*	32.67/0.19	6293	–	–	–
10	Emodin	*Rheum officinale*	83.38/0.24	3220	Wang S. et al., 2017	Hu et al., 2015	Yang et al., 2016
11	Aloe emodin	*Rheum officinale*	83.38/0.24	10207	–	Dou et al., 2018	–
12	Physcion 8-gentiobioside	*Rheum officinale*	41.65/0.63	442762	Pan et al., 2016	–	–
13	(-)-Maackiain	*Rheum officinale*	75.18/0.54	2035-15-6 (CAS)	Huang et al., 2018	–	–

*PubChem CID, compound identifier on PubChem database. CAS, Chemical Abstracts Service. DL, drug-likeness; OB, oral bioavailability. Drug screening criteria (OB ≥ 30% and DL ≥ 0.18) suggested by TCMSP database.*

### QDTS Granules Reduced Proteinuria of db/db Mice Effectively and Safely

**FIGURE 1:**
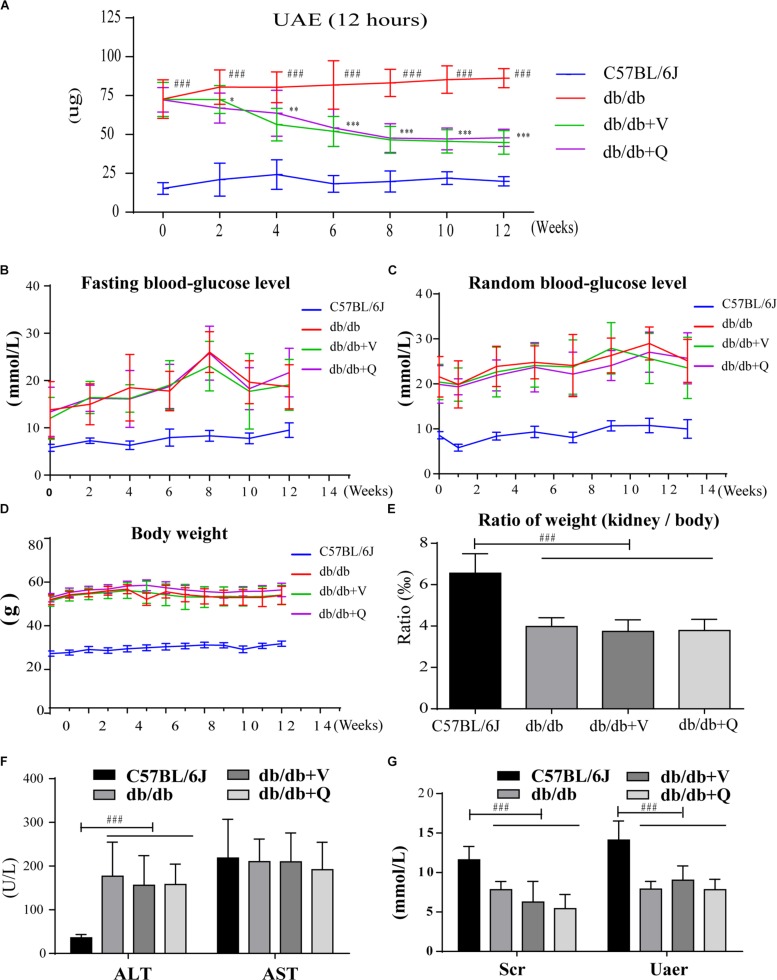
QDTS granules reduced 12 h UAE of db/db mice effectively and safely. **(A–C)** Describe changes in 12 h UAE, fasting blood glucose, and random blood glucose during drug intervention, respectively (detected once every 2 weeks). **(D,E)** Show the effects on body weight and the ratio of kidney weight to body weight. **(F,G)** Showed that QDTS did not cause liver and kidney dysfunction. ALT increased in db/db mice, which might be related to fatty liver. ^∗^*p* < 0.05, ^∗∗^*p* < 0.01, and ^∗∗∗^*p* < 0.001: Compared with the model group, respectively; ^###^*p* < 0.001: compared with the normal control group.

Between the normal control group and the db/db group, there were significant differences in the body weight, fasting blood glucose, random blood glucose, serum creatinine, urea nitrogen, ALT and ratio of kidney weight to body weight. However, there were no statistical differences in these indicators among three groups of db/db mice (Figures 1B–G). Before the drug intervention, all the db/db mice in three groups kept good living conditions and uniform hair color. Compared with the model group, 12 h UAE had been gradually and significantly alleviated since the second week of intervention and kept stabilization since the eighth week after treatment with valsartan or QDTS. (*p* < 0.05, Figure 1A).

### QDTS Granules Alleviated Renal Histological Injury

**FIGURE 2:**
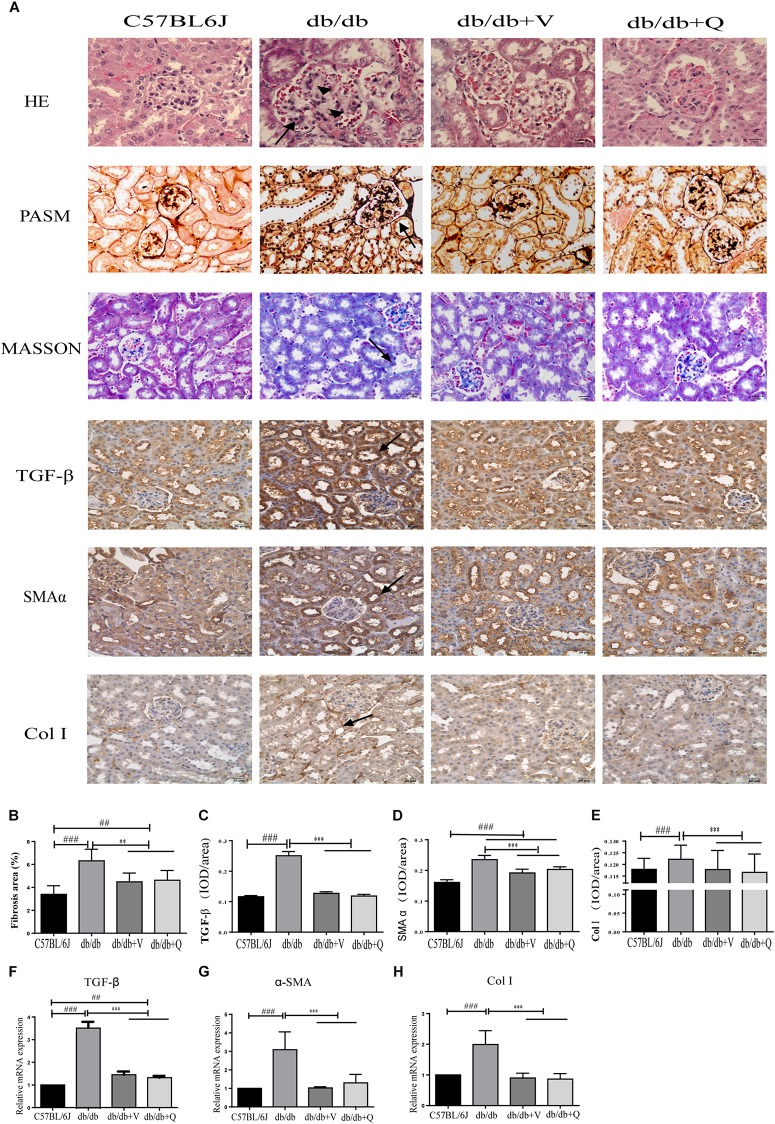
QDTS alleviated renal histological injury. **(A)** HE staining, Masson’s trichrome staining, PASM staining of renal tissues, and immunohistochemical staining for TGF-β, α-SMA, and Col I, respectively. **(B)** Degree of renal fibrosis. Imagine Pro Plus software was used to calculate results (the area of the blue area in each slice multiplied by its average optical density), which were compared between groups. **(C–E)** Are the statistic results of immunohistochemical staining of TGF-β, α-SMA, and Col I, respectively. The value of mean optical density (total optical density/total area) obtained by Imagine Pro Plus software. **(F–H)** Are the RT-qPCR statistic results of mRNA of TGF-β, α-SMA, and Col I, respectively. ^∗∗^*p* < 0.01 and ^∗∗∗^*p* < 0.001: compared with the model group, respectively; ^##^*p* < 0.01 and ^###^*p* < 0.001: compared with the normal control group, respectively. In the db/db group, glomerular mesangial hyperplasia (arrowheads) with mononuclear infiltration (arrow) was observed in HE staining, and the positive parts of other lesions are also marked with arrows.

**FIGURE 3:**
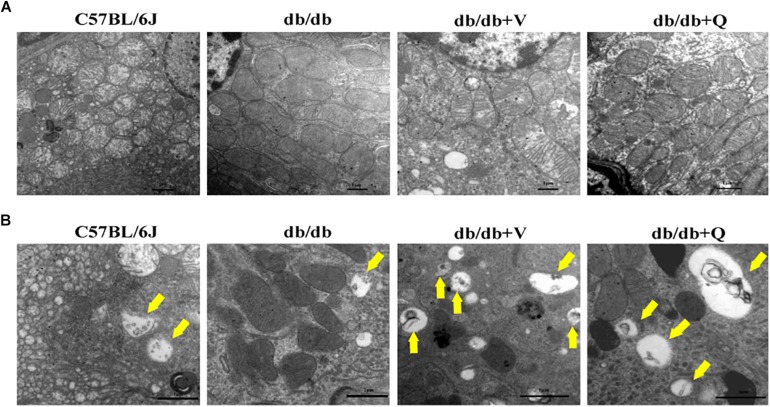
QDTS improved mitochondrial damage and autophagy in renal tubular epithelial cells. **(A)** Mitochondrial damage. Compared with the normal control group, massive mitochondria with incomplete boundary arranged tightly in the renal tubular epithelial cells of the db/db group, which meant that the damaged mitochondria accumulated and couldn’t be degraded timely; the blurred mitochondrial crista and disappeared double-layer membrane constructor implied that the ability of oxidative phosphorylation decreased (×40000). Valsartan and QDTS alleviated these damages of mitochondria to some extent. **(B)** Autophagic vacuoles. Compared with the normal control group, the number of autophagic vacuoles in the tubular epithelial cells of db/db mice was decreased, and the volume was relatively smaller (×70000). The number and volume of autophagic vacuoles in the valsartan and QDTS groups were significantly improved.

Compared to the normal control group, glomerular mesangial hyperplasia with mononuclear infiltration was observed in HE staining in the model group. PASM and MASSON staining showed that tubular brush border injury and tubulointerstitial fibrosis were obvious in db/db mice compared with the normal control. These pathological changes were significantly ameliorated by treatment with valsartan or QDTS granules (Figures 2A,B). Similarly, the results of the fibrosis-related indicators (TGF-β, α-SMA, and Col I) showed similar trends to the results of MASSON staining. That is, the expression of protein and mRNA of fibrosis markers in the model group were significantly increased compared with the normal group. Both valsartan and QDTS relieved the expression of these indicators to a large extent (Figures 2A,C–H). By transmission electron microscope, it was found that the number of autophagic vacuoles reduced, and the degree of damage to mitochondria was more serious in the renal tubular cells of the model group compared to the normal control group. Valsartan and QDTS had relatively improved the number of autophagic vacuoles and alleviated the mitochondria lesion (Figure 3).

### QDTS Granules Activated Renal Autophagy

**FIGURE 4:**
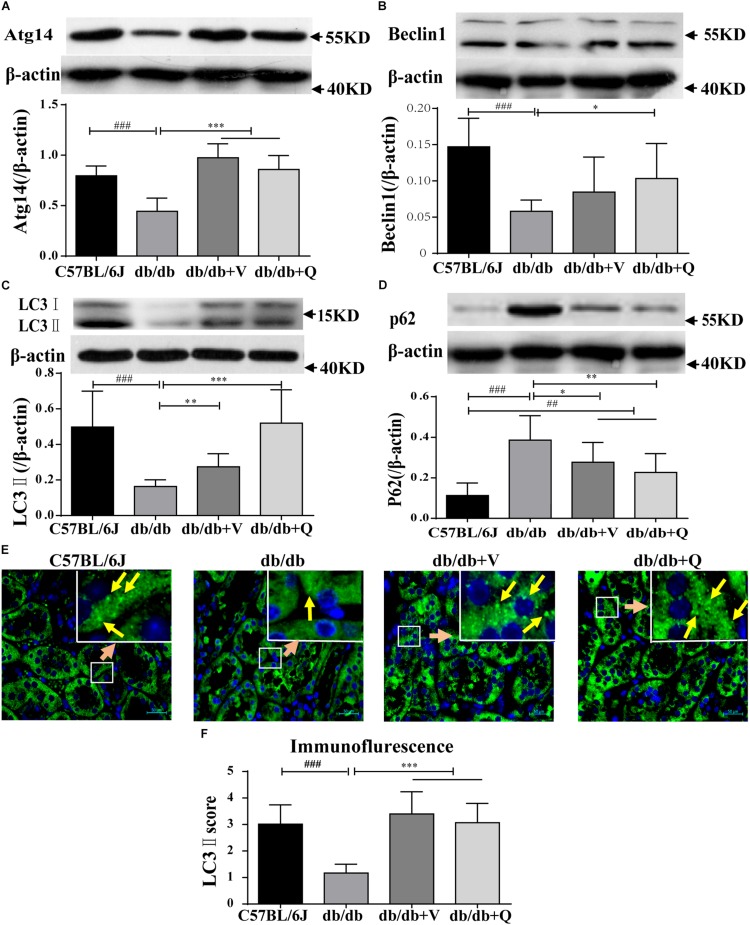
Renal autophagy| activity of db/db mice was activated by QDTS. **(A–D)** Are the expression of Atg14, Beclin1, LC3-II, and p62, respectively; **(E)** is the staining result (400x) of LC3-II immunofluorescence of paraffin sections. The small soft highlights in the figure are the specific expression of LC3-II, which was blindly reviewed and given a score by three pathologists, according to the quantity from least to greatest (score 0–5) divided into five levels. The results of evaluation are shown in **(F)**. ^∗^*p* < 0.05, ^∗∗^*p* < 0.01, and ^∗∗∗^*p* < 0.001: Compared with the model group, respectively; ^##^*p* < 0.01 and ^###^*p* < 0.001: compared with the normal control group, respectively.

We found that the expression of LC3-II was suppressed, while the autophagic substrate transporter p62 was up-regulated in the kidneys of db/db mice, compared with the normal control group. And the expression of Atg14 or beclin1 was lower in the model group than that in the normal control, suggesting inhibition of autophagic induction (Figures 4A–D). Interestingly, western blot analysis showed that QDTS and valsartan significantly promoted the autophagic induction, characterized by the increased expression of ATG14 and beclin1. The expression of LC3-II was up-regulated, but the protein level of p62 was down-regulated by QDTS or valsartan, indicating autophagic activation (Figures 4A–D). Similar results were acquired by the assay of immunofluorescence (Figure 4E). However, QDTS did not enhance the renal autophagic activity in normal control mice (Supplementary Figure S3).

### QDTS Granules Normalized the Expression of Nutrient-Sensing Signal Molecules

**FIGURE 5:**
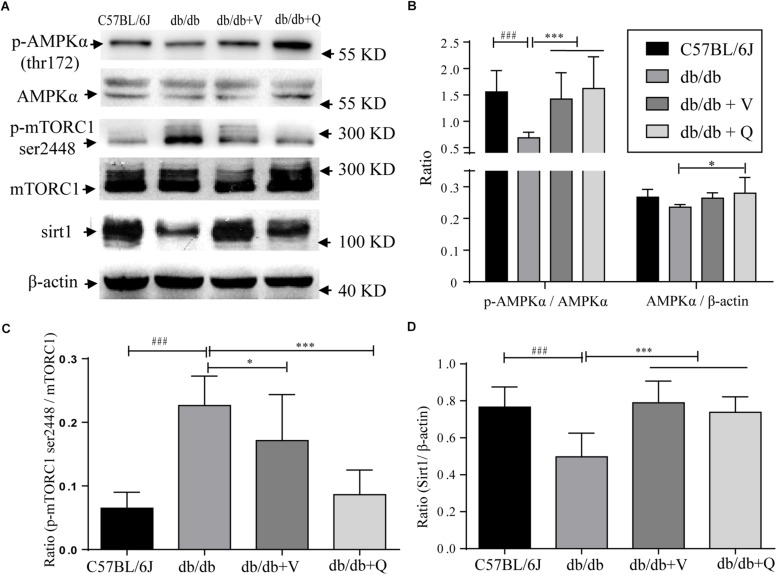
Nutrient-sensing signal molecules were ameliorated by QDTS. **(A)** Is a collection of protein bands. **(B)** Shows the effect of drug intervention on total AMPK protein and the level of p-AMPK thr172/AMPK in the kidney. **(C)** Shows the effect on the level of mTORC1 ser2448/mTORC1. **(D)** Indicates the expression of SIRT1 in the kidney. ^∗^*p* < 0.05 and ^∗∗∗^*p* < 0.001: Compared with the model group, respectively; ^###^*p* < 0.001: compared with the normal control group.

In the present study, we found that both the proportion of p-AMPK (ser172)/total AMPK and the expression of SIRT1 were down-regulated, while the renal proportion of p-mTORC1 (ser2448)/total mTOR was up-regulated in db/db mice compared with the normal control group. Either valsartan or QDTS normalized the expressions of these nutrient-sensing signal molecules in the kidneys of db/db mice (Figures 5A–C). Also, QDTS, but not valsartan improved the expression of total AMPK compared to the model group (Figure 5B).

## Discussion

It is believed that the deficiency of qi and essence (yin), as well as the unsmooth flow of blood, are the pathogenesis of DN in traditional Chinese medicine (Shi et al., 2018). The primary treatment strategy of QDTS is aimed at filling qi and yin and promoting blood circulation within the kidney (Hu et al., 2019). Astragalus and Rehmannia contribute to filling essence (yin) and qi of the kidney. Also, Euryale ferox and Cornus officinalis have the effect of solidifying and preventing the loss of kidney essence (yin). On the other hand, Rhubarb, Leeches and Hedyotis diffusa have the effects of dredging by smoothing, promoting blood circulation and clearing away heat and detoxification. Most herbs within QDTS, are widely used in the clinical treatment of chronic kidney disease in China, among which *Astragalus propinquus* is the most frequently used Chinese herb for treating DN (Liu et al., 2019). Although the effects of certain herbs within QDTS on reducing blood glucose have been reported (Liang et al., 2018), such effect of QDTS on db/db mice has not been found in this research and our previous study (Gao et al., 2018).

The regulation of the active components to the three nutrient signaling molecules was consistent with the effect of QDTS granules as a whole in the present research. AMPK, Sirt1, and mTOR, which are three crucial nutrient-sensing signals associated with autophagy, are receiving increasing attention in the study of DN. Their expressions are altered by excessive nutrient state of DN (Kume et al., 2012; Giovannini and Bianchi, 2017). Numerous studies referred to the regulation of every active component of QDTS to the three molecules were retrieved on Pubmed. It was found that among the 13 active components, eight components had been reported to activate AMPK, and six negatively regulated mTOR, while one positively regulated mTOR (Sun et al., 2018), as well as six up-regulated SIRT1 (Table 1 and Figure 5). Similar, our research also found that QDTS up-regulated the expression of SIRT1 and the proportion of both p-AMPK (thr172) to total AMPK, and down-regulated the ratio of p-mTOR (ser2448) to total mTOR.

QDTS attenuated renal histological lesions in db/db mice likely by activating renal autophagy. The accumulation of AGEs, activation of polyol metabolic bypass and oxidative stress, *etc.*, as typical pathological mechanisms of DN, cause intracellular accumulation of toxic metabolite wastes, mitochondrial damage, deposition of ECM and tubulointerstitial fibrosis in kidney by altering gene expression and protein function (Kitada et al., 2010). Autophagy plays a crucial role in maintaining cellular homeostasis by removing damaged mitochondria and metabolic wastes (Mizushima and Komatsu, 2011). The autophagy-associated protein and the autophagic substrate transporter p62 are essential for the synthesis of autophagic vacuoles and transportation of the substrate, separately. Impaired autophagy under the excessive nutrient state of DN, cannot remove AGEs, damaged mitochondria and metabolic wastes, causing renal histological lesions (Ding and Choi, 2015; Sifuentes-Franco et al., 2017). As the suppressed autophagic activity was alleviated by both QDTS and valsartan, the synthesis of Atg14, Beclin1 and LC3-II were increased (Figure 4), resulting in the degradation of autophagy substrate and cargos (such as damaged mitochondria, *etc.*) by autophagy. In addition, it was reported that AMPK and SIRT1 promoted quality control of mitochondria by promoting the synthesis of new mitochondria and the degradation of damaged mitochondria (Ou et al., 2014; Lucero et al., 2019). This meant that the autophagic degradation and regeneration balance of mitochondria was improved, and mitochondrial damage was alleviated (Figure 3). Also, improved autophagic activity helped to reduce renal fibrosis (Song et al., 2018). Therefore, renal autophagic suppression and subsequent changes are not only considered to be important roles in the initiation and progression of DN but also a promising therapeutic target for DN prevention (Higgins and Coughlan, 2014; Sakai et al., 2019). So, the improvement of renal autophagy activity likely plays a key role in reducing renal mitochondrial damage and anti-fibrosis.

The regulation of nutrient-sensitive signal pathways improved renal autophagic suppression in DN. AMPK, mTOR, and SIRT1 independently or synergistically regulate multiple pathophysiological processes such as cell energy metabolism, autophagy, apoptosis, and cell survival (Giovannini and Bianchi, 2017). mTOR, as one of the most critical autophagy-related nutrients-regulating signals in DN, is a serine-threonine protein kinase and composes two complexes 1, 2 (mTORC1 and mTORC2). It was reported that the phosphorylation of mTORC1 (ser2448) could inhibit autophagy initiating complex (Ding and Choi, 2015). In contrast, AMPK, as a central metabolic regulator related to energy, is also a serine/threonine-protein kinase and can stimulate autophagy. The phosphorylation of AMPKα thr172 can activate AMPK (Xiao et al., 2011). Activation of AMPK can promote autophagy by both phosphorylating autophagy-initiating complex directly and dissociating mTORC1 from the complex indirectly, etc. (Salminen and Kaarniranta, 2012). SIRT1, as a histone deacetylase, can promote autophagy by deacetylating multiple autophagy-related proteins such as Atg5/7/8 (Lee et al., 2008). Also, SIRT1 can activate AMPK, or inhibit mTORC1to boost autophagy (Wang et al., 2019). In addition, we found that QDTS significantly regulated the differential expression of several miRNAs, including mmu-miR-466i-3p, mmu-miR-6481 and mmu-miR-709 (data not shown). And SIRT1 mRNA might be their predicted target when studying the relationship in both miRNA-base and TargetScan7.1 databases. The nutrient-sensing molecules are likely regulated by the mRNAs, although it awaits further research. Nevertheless, it allows us to believe that QDTS likely improve renal autophagic activity by regulating nutrition-related signaling molecules.

In our study, some shortcomings left to be further improved. Firstly, db/db mice, as the model of type 2 DN with significant hyperglycemia and insulin resistance, are the excellent obesity model with the leptin receptor gene mutation, whose renal lesions is not severe (Kitada et al., 2016). Secondly, wild type mice, instead of db/m, was used as a normal control, since it was reported that wild type mice better reflected the normal state of miRNA expression, and wild type littermates were reported to be superior to db/m as normal controls when performing miRNA sequence analysis (Du et al., 2017). Thirdly, QDTS with multi-components might offer reno-protective effects by modulating multi-targets, which makes much difficulty to support the causal relationship between nutrient-sensing signaling molecules and kidney damage directly, since it is difficult to demonstrate a causal relationship by only blocking one pathway. Meanwhile, these nutrient-related signaling molecules are involved in many fundamental processes of cellular and biological metabolism, so blocking all these crucial molecules is likely not feasible for cell survival (Cetrullo et al., 2015). Fourthly, the settings of pharmacokinetic parameters (OB and DL) are based on the other research (Li et al., 2015) and the recommendation of TCMSP database^1^, so it is possible that some active components are missing, such as sterol in Rehmannia (less abundance in mass spectrometry due to instability during heating). In addition, the autophagic activity of different organs or cells may be different in various animal models and disease stages (Ding and Choi, 2015; Thorburn, 2018).

## Conclusion

QDTS exerts renoprotective effects in db/db mice independent of blood glucose control. It likely attributes to the regulation of autophagy-related nutrient-sensing signals.

## Data Availability Statement

All datasets generated for this study are included in the manuscript/Supplementary Files.

## Ethics Statement

The animal study was reviewed and approved by the Animal Welfare and Ethics Review Committee of Dongzhimen Hospital affiliated to Beijing University of Chinese Medicine (certificate number: 17-06).

## Author Contributions

HL and WL designed the protocol. XW and LZ performed the experiments and edited the manuscript. BJ and XZ directed and participated in the sample detection. CW and AA identified and analyzed the components of QDTS. XG, BJ, and ZY analyzed the data. XW wrote the manuscript, which was also edited by AA and ZY. All authors read and approved the final manuscript.

## Conflict of Interest

The authors declare that the research was conducted in the absence of any commercial or financial relationships that could be construed as a potential conflict of interest.

## Footnotes

1 http://lsp.nwu.edu.cn/load_intro.php?id=29

## Supplementary Material

FIGURE S1 Total ion chromatograms of QDTS granules. **(A)** MS characterization of the positive irons of QDTS. **(B)** MS characterization of the negative irons of QDTS. **(C)** The chemical constituents of QDTS granules identified in positive and negative ion modes. **(D)** The species of major components identified within the QDTS.

FIGURE S2 MS/MS spectrum and fragmentation pathway of representative components. **(A)** Kaempferol’s multistage mass spectrometry. **(B)** Fragmentation pathway and product ion spectrum of Kaempferol. The retention time of peak two is 20.19 min, and the ion peaks are m/z 285.04046 [M + H]−, and the elemental composition is estimated to be C15H10O6. Fragment ions such as m/z229, 213, 185, 169, 151, 135 are generated in MS/MS, wherein the fragment ions m/z151 and 135 are complementary ions produced by cleavage of flavonoid. The fragment ion m/z229 with a high abundance is formed by the continuous loss of CO by the quasi-molecular ion peak conforming to the cleavage characteristic of the flavonol compound. Therefore, the compound one is identified as Kaempferol by literature comparison. Similarly, Kaempferol’s multistage mass spectrometry and corresponding cleavage behavior are shown in **(C,D)**; astragaloside IV is **(E,F)**. Based on the analysis method above, 78 components in QDTS were identified in positive and negative ion modes (1–24: 24 flavonoids, 25–40: 16 terpenoids, 41–47: six triterpenoids, 48–62: 16 phenolic acids, 63–68: six coumarins or lignans, 69: one iridoid, 70: one alkaloid, 71: one disaccharide, 72: one saponins and 73–78: six other compounds, respectively).

FIGURE S3 QDTS did not enhance the renal autophagic activity of normal mouse. Twenty normal C57BL6J mice were randomly divided into two groups (10 in each group). Each group was given normal saline and QDTS for 7 days, once a day (dose as described in the manuscript). Three mice were randomly selected from each group to detect the expression levels of autophagy marker proteins LC3 and p62 in the kidney. As shown in the figure, there was no statistical difference between the two groups (*p* > 0.05).

TABLE S1 Components identified in QDTS granules. RT, retention time; all molecular formula presented errors lower than 5 ppm.

## References

[B1] Cetrullo S. D’Adamo S. Tantini B. Borzi R. M. Flamigni F. (2015). mTOR, AMPK, and Sirt1: key players in metabolic stress management. *Crit. Rev. Eukaryot. Gene Expr.* 25 59–75. 10.1615/CritRevEukaryotGeneExpr.2015012975 25955819

[B2] Che J. Liang B. Zhang Y. Wang Y. Tang J. Shi G. (2017). Kaempferol alleviates ox-LDL-induced apoptosis by up-regulation of autophagy via inhibiting PI3K/Akt/mTOR pathway in human endothelial cells. *Cardiovasc. Pathol.* 31 57–62. 10.1016/j.carpath.2017.08.001 28985493

[B3] Chen W. Chang B. Zhang Y. Yang P. Liu L. (2015). Rhein promotes the expression of SIRT1 in kidney tissues of type 2 diabetic rat (Chinese). *Xi Bao Yu Fen Zi Mian Yi Xue Za Zhi* 31 615–619. 10.13423/j.cnki.cjcmi.007349 25940287

[B4] Ding Y. Choi M. E. (2015). Autophagy in diabetic nephropathy. *J. Endocrinol.* 224 R15–R30. 10.1530/joe-14-0437 25349246 PMC4238413

[B5] Dou F. Liu Y. Liu L. Wang J. Sun T. Mu F. (2018). Aloe-Emodin ameliorates renal fibrosis via inhibiting PI3K/Akt/mTOR signaling pathway In Vivo and In Vitro. *Rejuvenation Res*. 22 218–229. 10.1089/rej.2018.2104 30215298

[B6] Du G. Xiao M. Zhang X. Wen M. Pang C. Jiang S. (2017). *Alpinia oxyphylla* Miq. extract changes miRNA expression profiles in db-/db- mouse kidney. *Biol. Res.* 50:9. 10.1186/s40659-017-0111-1 28249617 PMC5331689

[B7] Federation I. D. (2017). *IDF DIABETES ATLAS Eighth Edition 2017.* Available at: https://www.diabetesatlas.org/resources/2017-atlas.html (accessed January 18, 2019).

[B8] Gao X. Liu H. An Z. He Q. (2018). QiDiTangShen granules reduced diabetic kidney injury by regulating the phosphorylation balance of the tyrosine and serine residues of insulin receptor substrate 1. *Evid. Based Complement. Alternat. Med.* 2018:2503849. 10.1155/2018/2503849 30050584 PMC6046148

[B9] Gautam J. Khedgikar V. Kushwaha P. Choudhary D. Nagar G. K. Dev K. (2017). Formononetin, an isoflavone, activates AMP-activated protein kinase/beta-catenin signalling to inhibit adipogenesis and rescues C57BL/6 mice from high-fat diet-induced obesity and bone loss. *Br. J. Nutr.* 117 645–661. 10.1017/s0007114517000149 28367764

[B10] Giovannini L. Bianchi S. (2017). Role of nutraceutical SIRT1 modulators in AMPK and mTOR pathway: evidence of a synergistic effect. *Nutrition* 34 82–96. 10.1016/j.nut.2016.09.008 28063518

[B11] Gross J. L. de Azevedo M. J. Silveiro S. P. Canani L. H. Caramori M. L. Zelmanovitz T. (2005). Diabetic nephropathy: diagnosis, prevention, and treatment. *Diabetes Care* 28 164–176. 10.2337/diacare.28.1.164 15616252

[B12] Guo Z. Liao Z. Huang L. Liu D. Yin D. He M. (2015). Kaempferol protects cardiomyocytes against anoxia/reoxygenation injury via mitochondrial pathway mediated by SIRT1. *Eur. J. Pharmacol.* 761 245–253. 10.1016/j.ejphar.2015.05.056 26086862

[B13] Han F. Li K. Pan R. Xu W. Han X. Hou N. (2018). Calycosin directly improves perivascular adipose tissue dysfunction by upregulating the adiponectin/AMPK/eNOS pathway in obese mice. *Food Funct.* 9 2409–2415. 10.1039/c8fo00328a 29595858

[B14] Han Y. Liu H. Lou X. Miao G. Wang X. (2014). Method of combination of disease and syndrome in treating stage IV proteinuria in diabetic nephropathy of Qi-Yin deficiency. *J. Changchun Univ. Tradit. Chin. Med.* 30 903–905. 10.13463/j.cnki.Cczyy.2014.05.055

[B15] Higgins G. C. Coughlan M. T. (2014). Mitochondrial dysfunction and mitophagy: the beginning and end to diabetic nephropathy? *Br. J. Pharmacol.* 171 1917–1942. 10.1111/bph.12503 24720258 PMC3976613

[B16] Hu H. Sun W. Gu L. B. Tu Y. Liu H. (2015). Molecular mechanism of emodin on inhibiting autophagy induced by HBSS in renal tubular cells (Chinese). *Zhongguo Zhong Yao Za Zhi* 40 1965–1970. 26390657

[B17] Hu J. Liu H. Zhang X. (2019). Pathogenesis of diabetic nephropathy with essence depletion and collat Eral impediment (Chinese). *J. Beijing Univ. Tradit. Chin. Med.* 42 8–11.

[B18] Huang Y. Hao J. Tian D. Wen Y. Zhao P. Chen H. (2018). Antidiabetic activity of a flavonoid-rich extract from *Sophora davidii* (Franch.) Skeels in KK-Ay Mice via Activation of AMP-Activated Protein Kinase. *Front. Pharmacol.* 9:760. 10.3389/fphar.2018.00760 30061831 PMC6055046

[B19] Hwang J. S. Kang E. S. Han S. G. Lim D. S. Paek K. S. Lee C. H. (2018). Formononetin inhibits lipopolysaccharide-induced release of high mobility group box 1 by upregulating SIRT1 in a PPARdelta-dependent manner. *PeerJ* 6:e4208. 10.7717/peerj.4208 29312829 PMC5756453

[B20] Kishore L. Kaur N. Singh R. (2017). Distinct biomarkers for early diagnosis of diabetic nephropathy. *Curr. Diabetes Rev.* 13 598–605. 10.2174/1573399812666161207123007 27924722

[B21] Kitada M. Ogura Y. Koya D. (2016). Rodent models of diabetic nephropathy: their utility and limitations. *Int. J. Nephrol. Renovasc. Dis.* 9 279–290. 10.2147/ijnrd.S103784 27881924 PMC5115690

[B22] Kitada M. Ogura Y. Monno I. Koya D. (2017). Regulating autophagy as a therapeutic target for diabetic nephropathy. *Curr. Diab. Rep.* 17:53. 10.1007/s11892-017-0879-y 28593583

[B23] Kitada M. Zhang Z. Mima A. King G. L. (2010). Molecular mechanisms of diabetic vascular complications. *J. Diabetes Investig.* 1 77–89. 10.1111/j.2040-1124.2010.00018.x 24843412 PMC4008020

[B24] Kume S. Thomas M. C. Koya D. (2012). Nutrient sensing, autophagy, and diabetic nephropathy. *Diabetes* 61 23–29. 10.2337/db11-0555 22187371 PMC3237646

[B25] Lee I. H. Cao L. Mostoslavsky R. Lombard D. B. Liu J. Bruns N. E. (2008). A role for the NAD-dependent deacetylase Sirt1 in the regulation of autophagy. *Proc. Natl. Acad. Sci. U.S.A.* 105 3374–3379. 10.1073/pnas.0712145105 18296641 PMC2265142

[B26] Li Y. Wang J. Xiao Y. Wang Y. Chen S. Yang Y. (2015). A systems pharmacology approach to investigate the mechanisms of action of Semen Strychni and Tripterygium wilfordii Hook F for treatment of rheumatoid arthritis. *J. Ethnopharmacol.* 175 301–314. 10.1016/j.jep.2015.09.016 26386382

[B27] Liang M.-Y. Xu C.-K. Cuo J. (2018). Research progress on clinical application of hypoglycemic chinese medicine (Chinese). *World Latest Med. Inform.* 18 94–96. 10.19540/j.cnki.cjcmm.20170928.010 29376252

[B28] Liu B. Bai M. Miao M. (2019). Medication rules and factor analysis for traditional Chinese medicine treating diabetic kidney disease based on data mining (Chinese). *Chin. J. Mod. Appl. Pharm.* 7 781–785. 10.13748/j.cnki.issn1007-7693.2019.07.002

[B29] Liu H. Zhang X. (2016). Application of ZHANG Jingyue’s theory of genuine yin and vital essence on the treatment of diabetic nephropathy (Chinese). *J. Beijing Univ. Tradit. Chin. Med.* 39 5–9.

[B30] Liu W. J. Xie S. H. Liu Y. N. Kim W. Jin H. Y. Park S. K. (2012). Dipeptidyl peptidase IV inhibitor attenuates kidney injury in streptozotocin-induced diabetic rats. *J. Pharmacol. Exp. Ther.* 340 248–255. 10.1124/jpet.111.186866 22025647

[B31] Lucero M. Suarez A. E. Chambers J. W. (2019). Phosphoregulation on mitochondria: integration of cell and organelle responses. *CNS Neurosci. Ther.* 25 837–858. 10.1111/cns.13141 31025544 PMC6566066

[B32] Mizushima N. Komatsu M. (2011). Autophagy: renovation of cells and tissues. *Cell* 147 728–741. 10.1016/j.cell.2011.10.026 22078875

[B33] Ou X. Lee M. R. Huang X. Messina-Graham S. Broxmeyer H. E. (2014). SIRT1 positively regulates autophagy and mitochondria function in embryonic stem cells under oxidative stress. *Stem Cells* 32 1183–1194. 10.1002/stem.1641 24449278 PMC3991763

[B34] Pan X. Wang H. Tong D. Wang C. Sun L. Zhao C. (2016). Physcion induces apoptosis in hepatocellular carcinoma by modulating miR-370. *Am. J. Cancer Res.* 6 2919–2931. 28042511 PMC5199765

[B35] Rafacho B. P. Stice C. P. Liu C. Greenberg A. S. Ausman L. M. Wang X. D. (2015). Inhibition of diethylnitrosamine-initiated alcohol-promoted hepatic inflammation and precancerous lesions by flavonoid luteolin is associated with increased sirtuin 1 activity in mice. *Hepatobiliary Surg. Nutr.* 4 124–134. 10.3978/j.issn.2304-3881.2014.08.06 26005679 PMC4405419

[B36] Sakai S. Yamamoto T. Takabatake Y. Takahashi A. Namba-Hamano T. Minami S. (2019). Proximal tubule autophagy differs in type 1 and 2 diabetes. *J. Am. Soc. Nephrol.* 30 929–945. 10.1681/asn.2018100983 31040190 PMC6551771

[B37] Salminen A. Kaarniranta K. (2012). AMP-activated protein kinase (AMPK) controls the aging process via an integrated signaling network. *Ageing Res. Rev.* 11 230–241. 10.1016/j.arr.2011.12.005 22186033

[B38] Shi L. Yang Y. Han X. Xu X. Ni Q. (2018). Staged diagnosis and treatment of diabetic nephropathy (Chinese). *J. Tradit. Chin. Med.* 59 2057–2060. 10.13288/j.11-2166/r.2018.23.019

[B39] Sifuentes-Franco S. Pacheco-Moises F. P. Rodriguez-Carrizalez A. D. Miranda-Diaz A. G. (2017). The role of oxidative stress, mitochondrial function, and autophagy in diabetic polyneuropathy. *J. Diabetes Res.* 2017:1673081. 10.1155/2017/1673081 29204450 PMC5674726

[B40] Song S. Tan J. Miao Y. Li M. Zhang Q. (2017). Crosstalk of autophagy and apoptosis: involvement of the dual role of autophagy under ER stress. *J. Cell Physiol.* 232 2977–2984. 10.1002/jcp.25785 28067409

[B41] Song Y. Tao Q. Yu L. Li L. Bai T. Song X. (2018). Activation of autophagy contributes to the renoprotective effect of postconditioning on acute kidney injury and renal fibrosis. *Biochem. Biophys. Res. Commun.* 504 641–646. 10.1016/j.bbrc.2018.09.003 30205956

[B42] Sulaiman M. K. (2019). Diabetic nephropathy: recent advances in pathophysiology and challenges in dietary management. *Diabetol. Metab. Syndr.* 11:7. 10.1186/s13098-019-0403-4 30679960 PMC6343294

[B43] Sun H. Yin M. Qian W. Yin H. (2018). Calycosin, a phytoestrogen isoflavone, induces apoptosis of estrogen receptor-positive MG-63 osteosarcoma cells via the phosphatidylinositol 3-Kinase (PI3K)/AKT/Mammalian target of rapamycin (mTOR) pathway. *Med. Sci. Monit.* 24 6178–6186. 10.12659/msm.910201 30182951 PMC6134888

[B44] Takahashi A. Takabatake Y. Kimura T. Maejima I. Namba T. Yamamoto T. (2017). Autophagy inhibits the accumulation of advanced glycation end products by promoting lysosomal biogenesis and function in the kidney proximal tubules. *Diabetes* 66 1359–1372. 10.2337/db16-0397 28246295

[B45] Thorburn A. (2018). Autophagy and disease. *J. Biol. Chem.* 293 5425–5430. 10.1074/jbc.R117.810739 29191833 PMC5900754

[B46] Tu Y. Gu L. Chen D. Wu W. Liu H. Hu H. (2017). Rhein inhibits autophagy in rat renal tubular cells by regulation of AMPK/mTOR signaling. *Sci. Rep.* 7:43790. 10.1038/srep43790 28252052 PMC5333140

[B47] Varshney R. Varshney R. Mishra R. Gupta S. Sircar D. Roy P. (2018). Kaempferol alleviates palmitic acid-induced lipid stores, endoplasmic reticulum stress and pancreatic beta-cell dysfunction through AMPK/mTOR-mediated lipophagy. *J. Nutr. Biochem.* 57 212–227. 10.1016/j.jnutbio.2018.02.017 29758481

[B48] Wang C. Liu C. Wang M. Ma Q. Li Y. Wang T. (2018). UPLC-HRMS-Based plasma metabolomic profiling of novel biomarkers by treatment with KDZI in cerebral ischemia reperfusion rats. *Molecules* 23:E1315. 10.3390/molecules23061315 29849010 PMC6099697

[B49] Wang Q. Wang H. Jia Y. Ding H. Zhang L. Pan H. (2017). Luteolin reduces migration of human glioblastoma cell lines via inhibition of the p-IGF-1R/PI3K/AKT/mTOR signaling pathway. *Oncol. Lett.* 14 3545–3551. 10.3892/ol.2017.6643 28927111 PMC5588063

[B50] Wang S. Li X. Guo H. Yuan Z. Wang T. Zhang L. (2017). Emodin alleviates hepatic steatosis by inhibiting sterol regulatory element binding protein 1 activity by way of the calcium/calmodulin-dependent kinase kinase-AMP-activated protein kinase-mechanistic target of rapamycin-p70 ribosomal S6 kinase signaling pathway. *Hepatol. Res.* 47 683–701. 10.1111/hepr.12788 27492505

[B51] Wang W. Sun W. Cheng Y. Xu Z. Cai L. (2019). Role of sirtuin-1 in diabetic nephropathy. *J. Mol. Med.* 97 291–309. 10.1007/s00109-019-01743-7 30707256 PMC6394539

[B52] Wu C. Cao H. Zhou H. Sun L. Xue J. Li J. (2017). Research progress on the antitumor effects of rhein: literature review. *Anticancer Agents Med. Chem.* 17 1624–1632. 10.2174/1871520615666150930112631 26419468

[B53] Xiao B. Sanders M. J. Underwood E. Heath R. Mayer F. V. Carmena D. (2011). Structure of mammalian AMPK and its regulation by ADP. *Nature* 472 230–233. 10.1038/nature09932 21399626 PMC3078618

[B54] Yang D. Livingston M. J. Liu Z. Dong G. Zhang M. Chen J. K. (2018). Autophagy in diabetic kidney disease: regulation, pathological role and therapeutic potential. *Cell Mol. Life Sci.* 75 669–688. 10.1007/s00018-017-2639-1 28871310 PMC5771948

[B55] Yang T. Wang J. Pang Y. Dang X. Ren H. Liu Y. (2016). Emodin suppresses silica-induced lung fibrosis by promoting Sirt1 signaling via direct contact. *Mol. Med. Rep.* 14 4643–4649. 10.3892/mmr.2016.5838 27748907 PMC5102032

[B56] Yao J. N. Zhang X. X. Zhang Y. Z. Li J. H. Zhao D. Y. Gao B. (2019). Discovery and anticancer evaluation of a formononetin derivative against gastric cancer SGC7901 cells. *Invest. New Drugs* 10.1007/s10637-019-00767-7 [Epub ahead of print]. 30929157

[B57] Zhang R. Qin X. Zhang T. Li Q. Zhang J. Zhao J. (2018). Astragalus polysaccharide improves insulin sensitivity via AMPK activation in 3T3-L1 adipocytes. *Molecules* 23:2711. 10.3390/molecules23102711 30347867 PMC6222405

