# Supplementary figures and images for: QiDiTangShen Granules Activate Renal Nutrient-Sensing Associated Autophagy in db/db Mice
Source: Front Physiol. 2019 Oct 1;10:1224. doi: 10.3389/fphys.2019.01224 (PMC6779835; doi:10.3389/fphys.2019.01224)

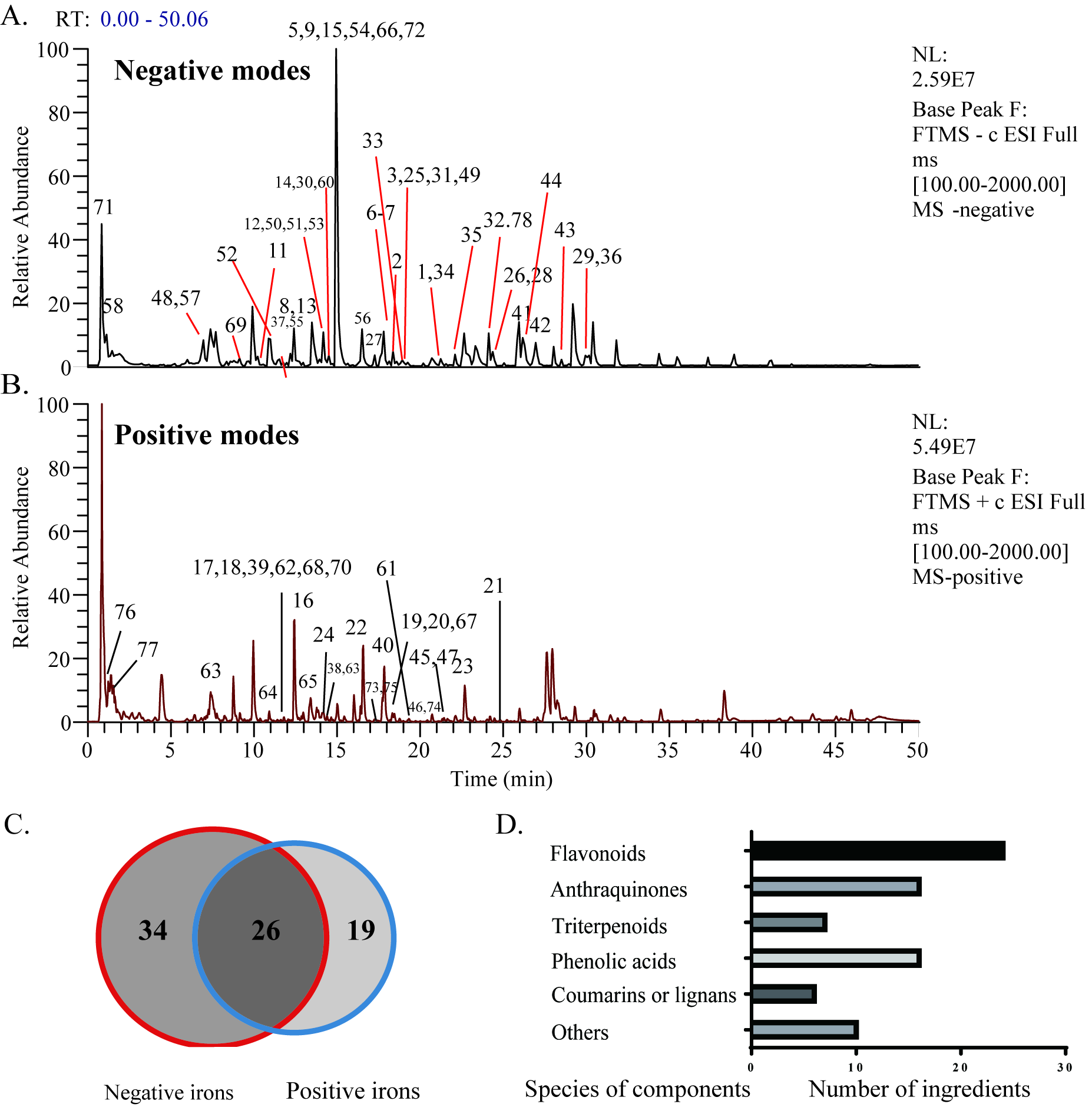

Supplement: FIGURE S1 — Total ion chromatograms of QDTS granules. (A) MS characterization of the positive irons of QDTS. (B) MS characterization of the negative irons of QDTS. (C) The chemical constituents of QDTS granules identified in positive and negative ion modes. (D) The species of major components identified within the QDTS. [file Image_1.TIF]

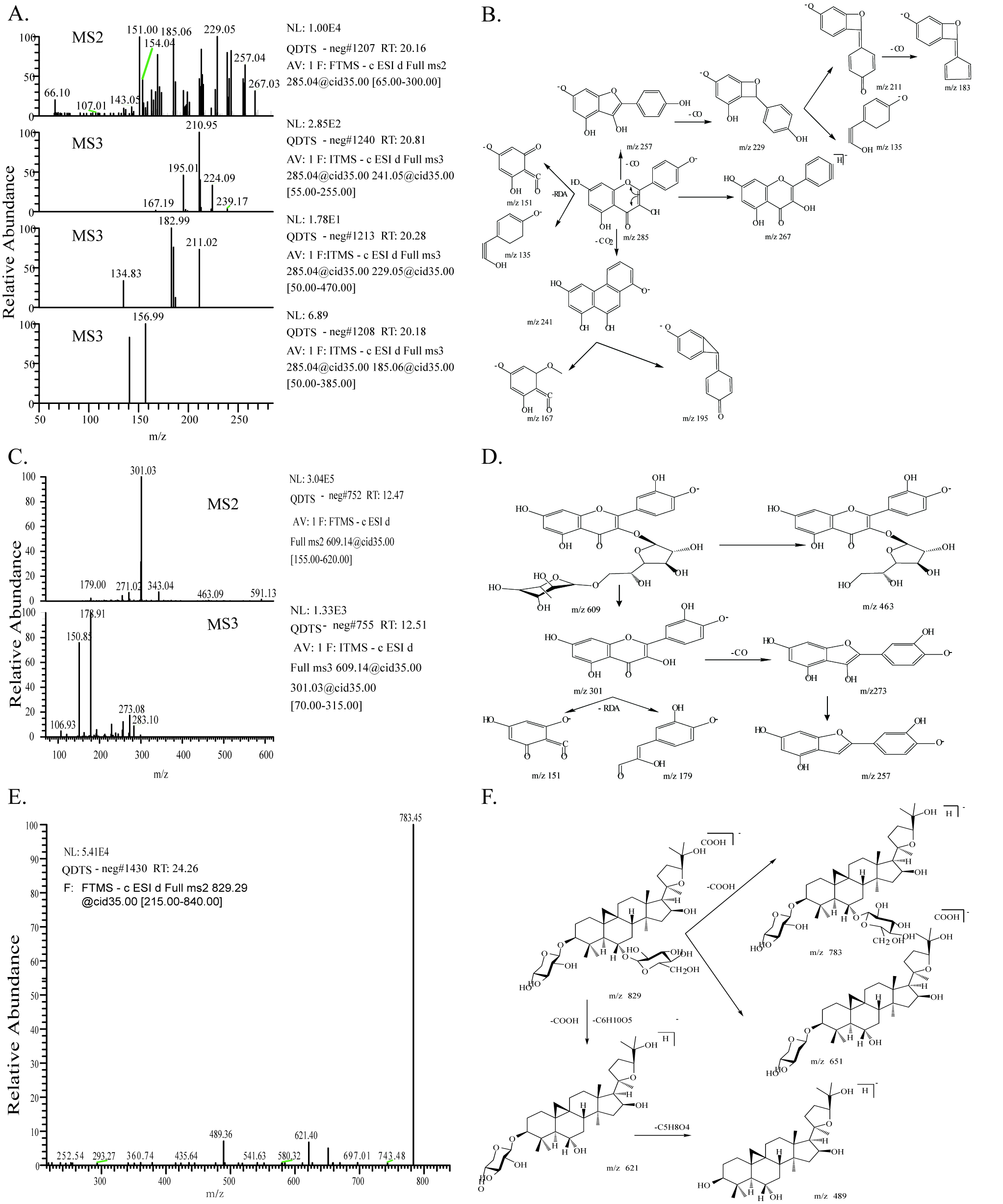

Supplement: FIGURE S2 — MS/MS spectrum and fragmentation pathway of representative components. (A) Kaempferol’s multistage mass spectrometry. (B) Fragmentation pathway and product ion spectrum of Kaempferol. The retention time of peak two is 20.19 min, and the ion peaks are m/z 285.04046 [M + H]−, and the elemental composition is estimated to be C15H10O6. Fragment ions such as m/z229, 213, 185, 169, 151, 135 are generated in MS/MS, wherein the fragment ions m/z151 and 135 are complementary ions produced by cleavage of flavonoid. The fragment ion m/z229 with a high abundance is formed by the continuous loss of CO by the quasi-molecular ion peak conforming to the cleavage characteristic of the flavonol compound. Therefore, the compound one is identified as Kaempferol by literature comparison. Similarly, Kaempferol’s multistage mass spectrometry and corresponding cleavage behavior are shown in (C,D); astragaloside IV is (E,F). Based on the analysis method above, 78 components in QDTS were identified in positive and negative ion modes (1–24: 24 flavonoids, 25–40: 16 terpenoids, 41–47: six triterpenoids, 48–62: 16 phenolic acids, 63–68: six coumarins or lignans, 69: one iridoid, 70: one alkaloid, 71: one disaccharide, 72: one saponins and 73–78: six other compounds, respectively). [file Image_2.TIF]

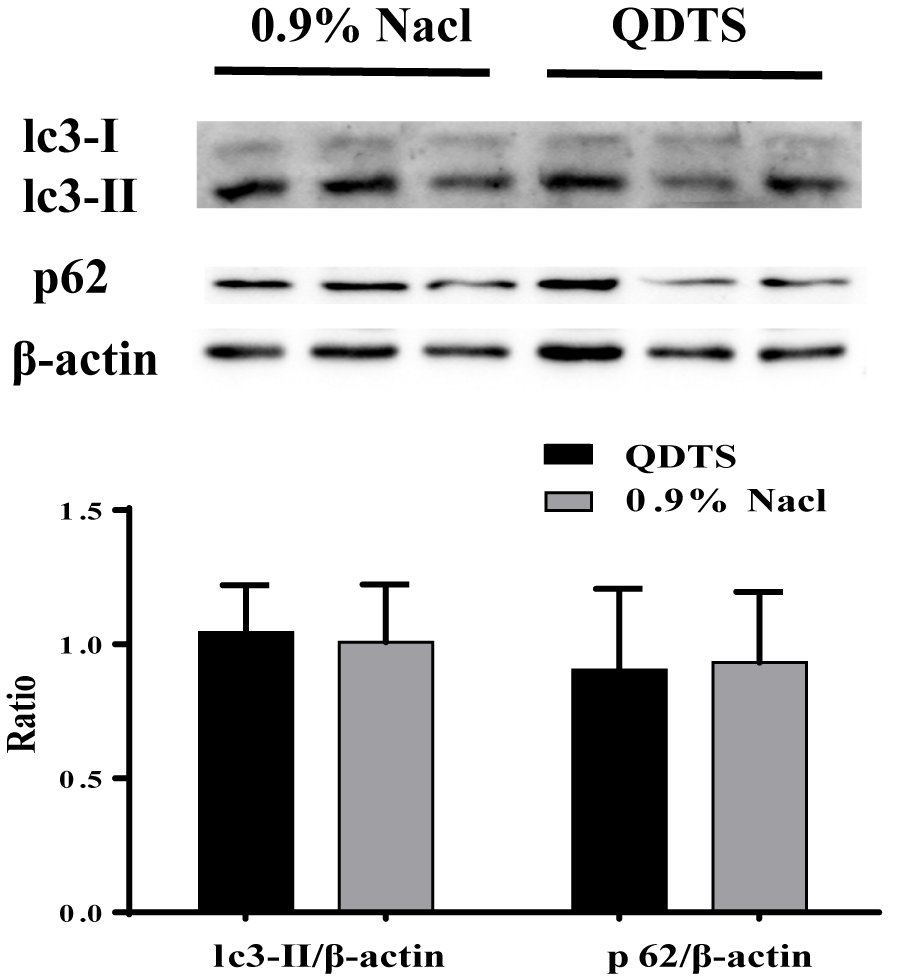

Supplement: FIGURE S3 — QDTS did not enhance the renal autophagic activity of normal mouse. Twenty normal C57BL6J mice were randomly divided into two groups (10 in each group). Each group was given normal saline and QDTS for 7 days, once a day (dose as described in the manuscript). Three mice were randomly selected from each group to detect the expression levels of autophagy marker proteins LC3 and p62 in the kidney. As shown in the figure, there was no statistical difference between the two groups (p > 0.05). [file Image_3.TIF]
