# Supplementary material for: QiDiTangShen Granules Activate Renal Nutrient-Sensing Associated Autophagy in db/db Mice
Source: Front Physiol. 2019 Oct 1;10:1224. doi: 10.3389/fphys.2019.01224 (PMC6779835; doi:10.3389/fphys.2019.01224)
Supplement: TABLE S1 — Components identified in QDTS granules. RT, retention time; all molecular formula presented errors lower than 5 ppm. [file Table_1.DOCX]

**Supplementary table 1.** Components identified in QDTS granules

| **Peak No.** | ***t*R/min** | **Molecul-ear formula** | **Detection pattern** | | ***m/z*** | **Secondary debris（MS/MS）** | **Identification** | **Source** | **OB / DL** | Number of Pubchem CID or CAS |  |
| --- | --- | --- | --- | --- | --- | --- | --- | --- | --- | --- | --- |
| 1 | 22.65 | C_16_H_12_O_4_ | - | 267.06628 | | 252.04,267.07,268.07 | Formononetin | Astragalus membranaceus | 69.67/0.21 | 5280378 |  |
| 2 | 20.19 | C_15_H_10_O_6_ | - | 285.04046 | | 135,151.00,169.07,185.06,213.06,229.05 | Kaempferol | Astragalus membranaceus | 41.88/0.24 | 5280863 |  |
| 3 | 20.81 | C_15_H_10_O_6_ | - | 285.04046 | | 257,241,211 | Luteolin | Astragalus membranaceus | 36.16/0.39 | [5280445](http://pubchem.ncbi.nlm.nih.gov/summary/summary.cgi?cid=5280445) |  |
| 4 | 17.15 | C_17_H_12_O_6_ | - | 311.05611 | | 223.17,293.21,275.20,235.17 | Acicerone | Astragalus membranaceus | - | [2746-86-3](http://www.ichemistry.cn/chemistry/2746-86-3.htm) |  |
| 5 | 15.65 | C_21_H_20_O_10_ | - | 431.09837 | | 311, 285, 269, 153 | Kaempferide 3-alpha-L-arabinopyranoside | Astragalus membranaceus | - | 123442-27-3 |  |
| 6 | 18.82 | C_21_H_20_O_10_ | - | 431.09837 | | 311, 269 | Isovitexin | Herba hedyotis | 31.29/0.72 | [162350](http://pubchem.ncbi.nlm.nih.gov/summary/summary.cgi?cid=162350) |  |
| 7 | 18.37 | C_23_H_28_O_10_ | - | 463.16097 | | 301.11,285.08 | Hyperin | Astragalus membranaceus | 6.94/0.77 | [5281643](http://pubchem.ncbi.nlm.nih.gov/summary/summary.cgi?cid=5281643) |  |
| 8 | 12.74 | C_21_H_20_O_12_ | - | 463.08820 | | 301.03,300.03,343.04 | Isomucronulator 7-O-glucoside | Astragalus membranaceus | - | 136087-29-1 |  |
| 9 | 16.50 | C_23_H_24_O_11_ | - | 475.12458 | | 267.07,312.06，429.12, | 7,2'-Dihydroxy-3',4'-dimethoxyisoflavone 7-O-glucoside | Astragalus membranaceus | - | 113235-89-5 |  |
| 10 | 10.99 | C_22_H_22_O_12_ | - | 477.10385 | | 301.03,313.06,169.01 | Rhamnetin 3-galactoside | Astragalus membranaceus | - | 12758-76-8 |  |
| 11 | 11.86 | C_22_H_22_O_12_ | - | 477.10385 | | 301 | Isorhamnetin 3-O-beta-D-glucopyranoside | Astragalus membranaceus | - | 5041-82-7 |  |
| 12 | 15.32 | C_27_H_30_O_15_ | - | 593.15119 | | 417.10,431.12,399.09 | Rhamnocitrin 3-apiosyl-(1->2)-glucoside | Astragalus membranaceus | - | 148031-68-9 |  |
| 13 | 12.41 | C_27_H_30_O_16_ | - | 609.14610 | | 301.03,343.04,271.02,255.03 | Bioquercetin | Astragalus membranaceus | - | 10371536 |  |
| 14 | 14.19 | C_28_H_32_O_16_ | - | 623.16176 | | 461.17,624.20，315.11,477.14 | Rhamnocitrin 3,4'-diglucoside | Astragalus membranaceus | - | 116183-66-5 |  |
| 15 | 15.21 | C_28_H_32_O_16_ | - | 623.16176 | | 461.17,315.11,477.14 | Isorhamnetin 3-O-beta-glucopyranoside-7-O-alpha-rhamnopyranoside | Astragalus membranaceus | - | 17331-71-4 |  |
| 16 | 12.81 | C_17_H_14_O_6_ | + | 315.08631 | | 300.05,283.02,255.08,301.01 | Kumatakenin | Astragalus membranaceus | 50.83/0.29 | [5318869](http://pubchem.ncbi.nlm.nih.gov/summary/summary.cgi?cid=5318869) |  |
| 17 | 12.41 | C_22_H_22_O_10_ | + | 447.12857 | | 285.10,285.95,270.22 | 7,5'-Hydroxy-3'-methoxyisoflavone 7-O-glucoside | Astragalus membranaceus | - | 241129-90-8 |  |
| 18 | 14.62 | C_22_H_22_O_11_ | + | 463.12349 | | 301.01,445.32 | Rhamnocitrin 3-glucoside | Astragalus membranaceus | - | 41545-37-3 |  |
| 19 | 18.51 | C_22_H_22_O_11_ | + | 463.12349 | | 301.01 | Kaempferide 3-O-beta-D-glucopyranoside | Astragalus membranaceus | - | [480-10-4](http://www.ichemistry.cn/chemistry/480-10-4.htm) |  |
| 20 | 18.47 | C_17_H_18_O_5_ | + | 303.12270 | | 166.96,148.93,122.83,180.92 | (-)-Mucronulatol | Astragalus membranaceus | 4.22/0.26 | [442811](http://pubchem.ncbi.nlm.nih.gov/summary/summary.cgi?cid=442811) |  |
| 21 | 24.78 | C_17_H_14_O_5_ | + | 299.09140 | | 284.03,266.00,238.06,285.03 | Afrormosin | Astragalus membranaceus | 23.54/0.27 | [5281704](http://pubchem.ncbi.nlm.nih.gov/summary/summary.cgi?cid=5281704) |  |
| 22 | 16.88 | C_17_H_14_O_5_ | + | 299.09140 | | 285,267,243 | 7-Hydroxy-3',5'-dimethoxyisoflavone | Astragalus membranaceus | - | 749849-58-9 |  |
| 23 | 23.22 | C_17_H_14_O_5_ | + | 285.07575 | | 284,267,243 | Calycosin | Rheum officinale | 47.75/0.24 | [5280448](http://pubchem.ncbi.nlm.nih.gov/summary/summary.cgi?cid=5280448) |  |
| 24 | 14.62 | C_16_H_12_O_6_ | + | 301.07066 | | 285.98,268.97,241.03,152.92 | Kaempferide | Astragalus membranaceus | 73.41/0.28 | [5281666](http://pubchem.ncbi.nlm.nih.gov/summary/summary.cgi?cid=5281666) |  |
| 25 | 19.04 | C_21_H_20_O_10_ | - | 431.09837 | | 311, 293, 269, 225 | 8-O-beta-D-glucopyranosyl emodin | Rheum officinale | - | 23313-21-5 |  |
| 26 | 25.06 | C_15_H_8_O_6_ | - | 283.02481 | | 239.03,257.04,146.97 | Rhein | Rheum officinale | 47.07/0.28 | [10168](http://pubchem.ncbi.nlm.nih.gov/summary/summary.cgi?cid=10168) |  |
| 27 | 17.59 | C_21_H_22_O_9_ | - | 417.11911 | | 254.06,255.07,297.08 | Cassialoin | Rheum officinale | - | 60462-09-1 |  |
| 28 | 25.06 | C_14_H_8_O_4_ | - | 239.03498 | | 211.04,183.04,212.04 | Alizarin | Rheum officinale | 32.67/0.19 | [6293](http://pubchem.ncbi.nlm.nih.gov/summary/summary.cgi?cid=6293) |  |
| 29 | 30.08 | C_15_H_10_O_5_ | - | 269.04555 | | 225.05,269.04,241.05 | Anthragallol 2-methyl ether | Herba hedyotis | - | 10383-63-8 |  |
| 30 | 14.51 | C_15_H_10_O_5_ | - | 269.04555 | | 269, 252, 239, 223, 195 | Emodin | Rheum officinale | 83.38/0.24 | [3220](http://pubchem.ncbi.nlm.nih.gov/summary/summary.cgi?cid=3220) |  |
| 31 | 19.25 | C_15_H_10_O_5_ | - | 269.04555 | | 225.05,269.04,241.05, 251.05 | Aloe emodin | Rheum officinale | 83.38/0.24 | [10207](http://pubchem.ncbi.nlm.nih.gov/summary/summary.cgi?cid=10207) |  |
| 32 | 23.32 | C_15_H_10_O_4_ | - | 253.05063 | | 253.05,254.05,225.05 | 3,7-Dihydroxyflavone | Astragalus membranaceus | - | 492-00-2 |  |
| 33 | 18.92 | C_15_H_10_O_4_ | - | 253.05063 | | 238, 223, 185 | Digiferrugineol | Herba hedyotis | 20.25/0.21 | [32209](http://pubchem.ncbi.nlm.nih.gov/summary/summary.cgi?cid=32209) |  |
| 34 | 22.78 | C_15_H_10_O_4_ | - | 253.05063 | | 238,224,181 | Rubiadin | Herba hedyotis | 25.02/0.21 | [124062](http://pubchem.ncbi.nlm.nih.gov/summary/summary.cgi?cid=124062) |  |
| 35 | 23.58 | C_15_H_10_O_4_ | - | 253.05063 | | 225,181 | Alizarin 1-methyl ether | Herba hedyotis | 8.70/0.21 | [80309](http://pubchem.ncbi.nlm.nih.gov/summary/summary.cgi?cid=80309) |  |
| 36 | 29.95 | C_15_H_10_O_4_ | - | 253.05063 | | 225,209 | Chrysophanol | Rheum officinale | 18.64/0.21 | [10208](http://pubchem.ncbi.nlm.nih.gov/summary/summary.cgi?cid=10208) |  |
| 37 | 12.74 | C_28_H_32_O_15_ | - | 607.16684 | | 413.11,193.05,431.12,588.21 | Physcion 8-gentiobioside | Rheum officinale | 41.65/0.63 | [442762](http://pubchem.ncbi.nlm.nih.gov/summary/summary.cgi?cid=442762) |  |
| 38 | 14.92 | C_11_H_10_O_3_ | + | 191.07027 | | 163.05,175.99,191.08,173.07 | 2,5-Dimethyl-7-hydroxychromone | Rheum officinale | - | [38412-47-4](http://www.ichemistry.cn/chemistry/38412-47-4.htm) |  |
| 39 | 12.44 | C_16_H_12_O_5_ | + | 285.07575 | | 270,253,229,225,137 | 2,3-Dimethoxy-9-hydroxy-1,4-anthraquinone | Herba hedyotis | - | 184652-26-4 |  |
| 40 | 17.85 | C_16_H_12_O_5_ | + | 285.07575 | | 270,253,229,225,137 | Physcion | Rheum officinale | 22.29/0.27 | [10639](http://pubchem.ncbi.nlm.nih.gov/summary/summary.cgi?cid=10639) |  |
| 41 | 26.73 | C_47_H_76_O_17_ | - | 911.50097 | | 893.49，571.40,457.37,615.39,525.39 | Nudicaucin B | Herba hedyotis | - | 211557-36-7 |  |
| 42 | 27.13 | C_46_H_76_O_18_ | - | 915.49589 | | 869.49,809.46,767.45,749.44,857.52,293.48 | Trojanoside B | Astragalus membranaceus | - | 223924-11-6 |  |
| 43 | 28.04 | C_46_H_76_O_18_ | - | 915.49589 | | 869, 857, 809, 767, 749, 587,525 | Trojanoside H | Astragalus membranaceus | - | 244630-00-0 |  |
| 44 | 26.85 | C_48_H_78_O_18_ | - | 941.51154 | | 923.50,526.39,733.45,615.39 | Soyasaponin I | Astragalus membranaceus | \| 2.06/0.27 \| \| --- \| | [122097](http://pubchem.ncbi.nlm.nih.gov/summary/summary.cgi?cid=122097) |  |
| 45 | 21.08 | C_47_H_78_O_19_ | + | 947.52101 | | 437.24,929.52，785.38，859.81 | Trojanoside K | Astragalus membranaceus | - | 386273-44-5 |  |
| 46 | 20.59 | C_47_H_78_O_19_ | + | 947.52101 | | 437.24,929.52，785.38，859.81 | Astragaloside VII | Astragalus membranaceus | - | 84687-46-7 |  |
| 47 | 20.70 | C_30_H_48_O_4_ | + | 473.36254 | | 269.07,347.45 | 3beta,24-Dihydroxyurs-12-en-28-oic acid | Herba hedyotis | - | 151214-01-6 |  |
| 48 | 7.49 | C_20_H_22_O_9_ | - | 405.11910 | | 243.09,373.11,179.06，155.03 | Astringin | Rheum officinale | 72.29/0.13 | [667639](http://pubchem.ncbi.nlm.nih.gov/summary/summary.cgi?cid=667639) |  |
| 49 | 18.93 | C_15_H_14_O_3_ | - | 241.08701 | | 226.06,225.06,197.06 | 3,5-Dihydroxy-4'-methoxystilbene | Rheum officinale | - | [30197-14-9](http://www.ichemistry.cn/chemistry/30197-14-9.htm) |  |
| 50 | 13.94 | C_21_H_24_O_9_ | - | 419.13476 | | 257.08,345.05,383.20 | Rhaponticin | Rheum officinale | - | 155-58-8 |  |
| 51 | 13.27 | C_23_H_26_O_11_ | - | 477.14023 | | 301.03,313.06,169.01 | Lindleyin | Rheum officinale | - | 59282-56-3 |  |
| 52 | 11.10 | C_35_H_46_O_20_ | - | 785.25096 | | 623.22,461.16,605.21 | Purpureaside C/ Purpureaside B | Radix Rehmanniae Praeparata | 3.14/0.38 | 82854-37-3 |  |
| 53 | 13.94 | C_15_H_14_O_4_ | - | 257.08193 | | 241.05,224.05,172.05 | Rhapontigenin | Rheum officinale | - | 500-65-2 |  |
| 54 | 15.10 | C_30_H_38_O_15_ | - | 637.21379 | | 461.17,491.15,475.18 | Jionoside D | Radix Rehmanniae Praeparata | 1.56/0.68 | 120406-34-0 |  |
| 55 | 12.19 | C_36_H_48_O_20_ | - | 799.26662 | | 623.22,605.21，461.16 | Jionoside A2 | Radix Rehmanniae Praeparata | 3.62/0.36 | 120406-36-2 |  |
| 56 | 17.27 | C_31_H_40_O_15_ | - | 651.22944 | | 475.18,505.17,457.17,193.05 | Martynoside | Radix Rehmanniae Praeparata | 12.91/0.58 | [5319292](http://pubchem.ncbi.nlm.nih.gov/summary/summary.cgi?cid=5319292) |  |
| 57 | 7.06 | C_9_H_8_O_4_ | - | 179.03498 | | 135.05,151.04,52.50 | Caffeic acid | Astragalus membranaceus | 54.97/0.05 | [1549111](http://pubchem.ncbi.nlm.nih.gov/summary/summary.cgi?cid=1549111) |  |
| 58 | 1.13 | C_4_H_4_O_4_ | - | 115.00368 | | 115.00,71.01,73.77 | Fumaric acid | Astragalus membranaceus | 17.74/0.01 | [21883788](http://pubchem.ncbi.nlm.nih.gov/summary/summary.cgi?cid=21883788) |  |
| 59 | 13.49 | C_29_H_36_O_15_ | - | 623.19814 | | 461.17,623.20,315.11 | Forsythiaside | Radix Rehmanniae Praeparata | 3.05/0.61 | [5281773](http://pubchem.ncbi.nlm.nih.gov/summary/summary.cgi?cid=5281773) |  |
| 60 | 14.18 | C_29_H_36_O_15_ | - | 623.19814 | | 461 | Acteoside | Radix Rehmanniae Praeparata | 2.94/0.62 | [5281800](http://pubchem.ncbi.nlm.nih.gov/summary/summary.cgi?cid=5281800) |  |
| 61 | 19.45 | C_14_H_12_O_4_ | + | 245.08084 | | 202.92,227.04,199.02,190.94,217.07 | Piceatannol | Rheum officinale | 72.29/0.13 | [667639](http://pubchem.ncbi.nlm.nih.gov/summary/summary.cgi?cid=667639) |  |
| 62 | 11.85 | C_29_H_36_O_13_ | + | 593.22287 | | 431.19,563.21,575.22,351.06 | Jionoside C | Radix Rehmanniae Praeparata | - | 120406-33-9 |  |
| 63 | 7.26 | C_16_H_18_O_9_ | + | 355.10236 | | 323.14,337.13,305.14，338.16 | Scopolin | Astragalus membranaceus | 22.91/0.39 | [25791637](http://pubchem.ncbi.nlm.nih.gov/summary/summary.cgi?cid=25791637) |  |
| 64 | 11.34 | C_10_H_8_O_4_ | + | 193.04954 | | 132.84,164.87,148.88,177.91 | Scopoletin | Astragalus membranaceus | 27.77/0.08 | [5280460](http://pubchem.ncbi.nlm.nih.gov/summary/summary.cgi?cid=5280460) |  |
| 65 | 13.53 | C_9_H_6_O_3_ | + | 163.03897 | | 145,135, 117,107 | 7-Hydroxycoumarin | Astragalus membranaceus | 27.37/0.05 | [5281426](http://pubchem.ncbi.nlm.nih.gov/summary/summary.cgi?cid=5281426) |  |
| 66 | 14.51 | C_22_H_26_O_8_ | - | 417.15549 | | 254.06,253.05,255.07 | (+)-Lirioresinol B | Astragalus membranaceus | - | 21453-69-0 |  |
| 67 | 17.73 | C_17_H_16_O_5_ | + | 301.10705 | | 166.91,191.08,146.90,269.01 | Astraciceran | Rheum officinale | - | 72026-91-6 |  |
| 68 | 12.42 | C_16_H_12_O_5_ | + | 285.07575 | | 270.02,252.97，225.00,136.89,271.00 | (-)-Maackiain | Rheum officinale | 75.18/0.54 | 2035-15-6 |  |
| 69 | 10.56 | C_16_H_20_O_10_ | - | 371.09837 | | 249.06,231.05,353.09,209.08 | Deacetyl asperuloside | Herba hedyotis | 15.27/0.54 | 18843-01-1 |  |
| 70 | 12.04 | C_21_H_2_6N_2_O_2_ | + | 339.20670 | | 176.91,321.09,144.91,194.95 | (+)-Chrysotricine | Herba hedyotis | - | 200884-09-9 |  |
| 71 | 0.83 | C_12_H_22_O_11_ | - | 341.10893 | | 179.06,342.11,143.03,161.05,113.02 | Sucrose | Astragalus membranaceus | 7.17/0.23 | [5988](http://pubchem.ncbi.nlm.nih.gov/summary/summary.cgi?cid=5988) |  |
| 72 | 14.73 | C_29_H_38_O_15_ | - | 625.21379 | | 462.17,463.17,177.37 | (3R)-7,2'-Dihydroxy-3',4'-dimethoxyisoflavan 7,2'-O-diglucoside | Astragalus membranaceus | - | 137217-84-6 |  |
| 73 | 17.62 | C_16_H_14_O_6_ | + | 303.08631 | | 257.08，228.94,164.94,285.04,246.98 | Carpusin | Rheum officinale | - | 87582-99-8 |  |
| 74 | 20.35 | C_17_H_16_O_6_ | + | 317.10196 | | 134.92,289.04,162.93,299.06 | Astragaluquinone | Astragalus membranaceus | - | 158991-20-9 |  |
| 75 | 17.13 | C_23_H_26_O_10_ | + | 463.15987 | | 301.05,400.07,445.37 | Methylnissolin 3-O-glucoside | Astragalus membranaceus | - | 94367-42-7 |  |
| 76 | 1.20 | C_9_H_17_NO_8_ | + | 268.10269 | | 135.90,181.98,250.08 | Miserotoxin | Astragalus membranaceus | - | 24502-76-9 |  |
| 77 | 1.94 | C_6_H_6_O_3_ | + | 127.03897 | | 98.79,108.87,70.79,80.77 | 5-Hydroxymethyl-2-furaldehyde | Radix Rehmanniae Praeparata | 45.07/0.02 | [237332](http://pubchem.ncbi.nlm.nih.gov/summary/summary.cgi?cid=237332) |  |
| 78 | 24.50 | C_41_H_68_O_14_ | - | 829.45764 | | 783.45,651.41,621.39,489.35,435.64,293.27 | Astragaloside IV | Astragalus membranaceus | 22.50/0.15 | 84687-43-4 |  |
